# Integrative Computational Analysis of Common EXO5 Haplotypes: Impact on Protein Dynamics, Genome Stability, and Cancer Progression

**DOI:** 10.1021/acs.jcim.5c00067

**Published:** 2025-03-21

**Authors:** Fabio Mazza, Davide Dalfovo, Alessio Bartocci, Gianluca Lattanzi, Alessandro Romanel

**Affiliations:** † Department of Cellular, Computational and Integrative Biology (CIBIO), University of Trento, Via Sommarive 9, Trento 38123, Italy; ‡ Department of Physics, University of Trento, Via Sommarive 9, Trento 38123, Italy; § INFN-TIFPA, Trento Institute for Fundamental Physics and Applications, Via Sommarive 14, Trento 38123, Italy

## Abstract

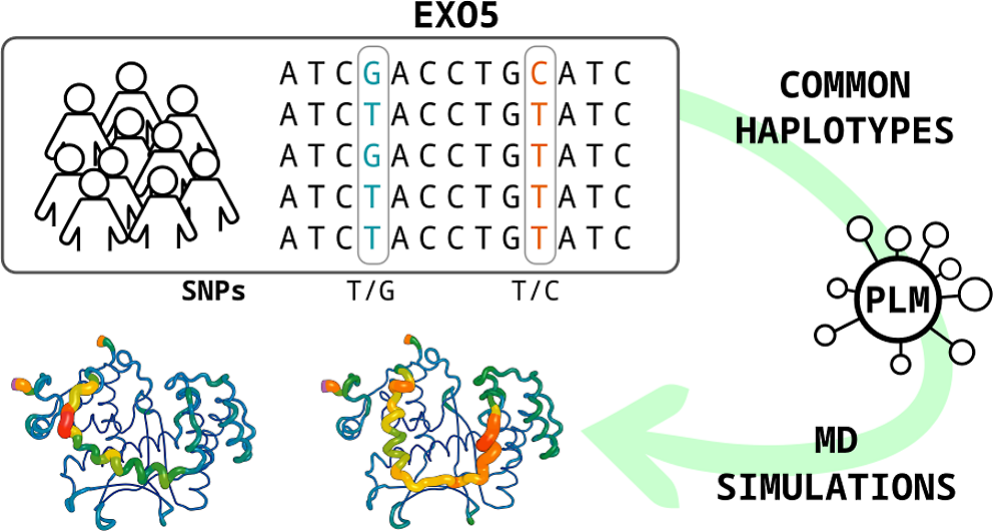

Understanding the impact of common germline variants on protein structure, function, and disease progression is crucial in cancer research. This study presents a comprehensive analysis of the *EXO5* gene, which encodes a DNA exonuclease involved in DNA repair that was previously associated with cancer susceptibility. We employed an integrated approach combining genomic and clinical data analysis, deep learning variant effect prediction, and molecular dynamics (MD) simulations to investigate the effects of common *EXO5* haplotypes on protein structure, dynamics, and cancer outcomes. We characterized the haplotype structure of *EXO5* across diverse human populations, identifying five common haplotypes, and studied their impact on the EXO5 protein. Extensive, all-atom MD simulations revealed significant structural and dynamic differences among the EXO5 protein variants, particularly in their catalytic region. The L151P EXO5 protein variant exhibited the most substantial conformational changes, potentially disruptive for EXO5′s function and nuclear localization. Analysis of The Cancer Genome Atlas data showed that cancer patients carrying L151P EXO5 had significantly shorter progression-free survival in prostate and pancreatic cancers and exhibited increased genomic instability. This study highlights the strength of our methodology in uncovering the effects of common genetic variants on protein function and their implications for disease outcomes.

## Introduction

DNA double-strand breaks (DSBs) represent one of the most severe forms of genetic damage, posing a significant threat to genomic stability and cellular health. If left unrepaired or improperly addressed, DSBs can lead to genomic instability, potentially disrupting oncogens and tumor suppressor genes, thereby increasing cancer susceptibility and impacting cancer evolution.^1−3^ Two primary DNA damage response (DDR) pathways tackle DSBs: homology-directed repair (HDR) and nonhomologous end joining (NHEJ). The choice between these pathways is governed by various factors, including cell cycle checkpoints and activation of specific DNA repair genes. Germline genetic variants are the primary form of DNA polymorphism and variants in genes encoding proteins involved in DDR have been shown to contribute not only to cancer susceptibility but also to play a crucial role in determining treatment response and clinical outcomes.^4−6^ While some major DNA repair mechanisms have been structurally characterized, a comprehensive understanding of many pathways, including the impact of common genetic variation on protein structure and dynamics, remains elusive. In particular, the use of molecular dynamics (MD) simulations to probe these dynamic aspects has been limited, leaving a gap in our understanding of how these variations might influence DNA repair at the molecular level. This gap is particularly relevant for proteins like specialized exonucleases, which carry out the crucial process of DNA end resection, a determinant of the chosen repair pathway.^7−9^

Among those, EXO5 is a single-stranded DNA (ssDNA) exonuclease implicated in DNA repair and is expressed by the *EXO5* gene. It is involved in homologous recombination following interstrand cross-links damage, specifically in the process of stalled DNA replication fork restart, where it performs DNA end resection.^10−12^ EXO5 loads at ssDNA ends, with 5′ to 3′ polarity enforced by replication protein A, and slides along ssDNA prior to the resection.^10,11^ It plays a role in managing stalled replication forks, but its precise function in this process is not fully understood.

Ali et al.^11^ identified *EXO5* as a risk gene for prostate cancer (PCa). They reported multiple *EXO5*-related germline single nucleotide variants [single nucleotide polymorphisms (SNPs)] associated with PCa risk and demonstrated that knockout of the *EXO5* gene leads to reduced HDR efficiency. Interestingly, the authors observed through in vitro experiments that a mutation of residue 151 from leucine to proline, as a result of the common germline SNP rs35672330, causes a loss of EXO5 nuclease activity and of nuclear localization.

The role of EXO5 in cancer was further characterized in ref (12) where the authors observed that elevated EXO5 expression in tumors correlates with increased mutation loads and poor patient survival, suggesting that *EXO5* upregulation has oncogenic potential. The authors hypothesized that high EXO5 levels may contribute to mutations by potentially shunting repair of replication errors away from error-free homologous recombination and into more error-prone pathways. Structural data generated in the same study indicate that the EXO5 channel where ssDNA inserts might contain a conditionally folded region, composed of an α helix (α4) that unfolds upon DNA insertion. Further, the authors observed that without the presence of an iron–sulfur cluster bonded near the N-terminus of the protein, the nuclease function is mostly absent. The catalytic mechanism uses either a one-metal ion or a two-metal ion, and without availability of divalent cations such as magnesium ions, the catalytic activity is also absent.

Although the role of EXO5 in cancer appears to be important for both tumor initiation and progression, comprehensive structural studies remain lacking. Specifically, while the structural biology of major DNA repair mechanisms has received some attention, the influence of common genetic variations on the structure and dynamics of DNA repair proteins has often been overlooked. Furthermore, most of these DNA repair mechanisms have not been explored through the lens of MD, which can offer critical insights into protein dynamics and function. In the case of EXO5, there has been no investigation into how common germline genetic variants might alter the structural conformation and dynamic properties of the protein, which could have implications for its function in DNA repair pathways and its potential as a therapeutic target.

To address these knowledge gaps, here we utilized an integrated approach that combines the interrogation of large genomic data sets with deep learning predictions and MD simulations. Initially, we assess and characterize common human *EXO5* haplotypes using the ESM-1v pretrained protein language model (PLM).^13,14^ Following this, we conduct multiple 3 ms long MD simulations to understand how the predicted fitness differences influence the protein’s structure and dynamics. Finally, we demonstrate the prognostic significance of the most impactful haplotype by analyzing cancer data from The Cancer Genome Atlas (TCGA).

Overall, our strategy enabled us to both explore how common human haplotypes affect the structural and dynamic properties of the EXO5 protein and identify potential genetic biomarkers for stratifying cancer patients according to distinct clinical outcomes. Extensive MD simulations provide a mechanistic understanding of the structural variations among different EXO5 protein variants. These insights not only validate the predictions of deep learning models but also enrich them with detailed mechanistic interpretations.

## Materials and Methods

### Identification of Transcript-specific Haplotypes

Phased 1000 Genomes Project SNP genotypes were downloaded (www.internationalgenome.org/) and annotated using SnpEff v5.1d.^15^ Variants annotated as Missense were retrieved and combined with the human reference genome (hg38) to build the landscape of alleles for all protein-coding transcripts of *EXO5* and a set of DDR genes of interest.^16^ Haplotype frequencies were then computed for each transcript across all 1000 Genomes Project individuals, and all haplotypes showing a frequency greater than 0.01 were selected.

### Calculation of Haplotypes’ Functional Scores

To quantify the effect of germline SNPs within haplotypes on protein structures, we utilized the evolutionary scale modeling ESM-1v PLM.^13^ We use the pseudo log-likelihood ratio (PLLR) score^17^ to quantify this impact, building on the work presented in^18^
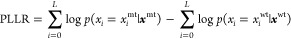


The PLLR score quantifies the difference between the model’s pseudo log-likelihood of a mutated protein sequence—specifically, one that incorporates changes introduced by haplotype-specific germline SNPs—and its corresponding wild-type protein sequence, by calculating their logarithmic difference. The log-likelihood, in this context, represents how likely a sequence is according to the model’s interpretation of protein grammar, which we estimate by summing the log-probabilities of amino acids as returned by the model’s output.

A positive PLLR score indicates that the variants make the sequence more plausible or less disruptive according to the model, while a negative PLLR score suggests a potentially harmful impact, implying that the variants may result in a sequence that is less likely to be functional or stable. More specifically, we compute the PLLR between SNP-containing sequences and their respective reference transcript sequence using the ensemble of ESM-1v models 1 to 3. These correspond to the same ESM architecture with the same training data but with different starting seeds for their training. We then consider the averages of the three resulting PLLR values for each nonreference sequence.

### MD Simulations

The impact of *EXO5* haplotypes on the structural conformation and dynamics of the EXO5 protein was assessed through multireplica, all-atom MD simulations.^19^

#### Reconstruction and Modeling of EXO5 Protein Structures

The 7LW9 crystallographic structure of EXO5^12^ was obtained from the RCSB protein data bank. The 7LW9 PDB file includes 255 modeled amino acids of the G172V variant of EXO5, with 118 residues missing, along with a seven-nucleotide single-stranded DNA molecule (TGAAGGG) and an iron–sulfur cluster [4Fe–4S]. The sequence and secondary structure of the 7LW9 PDB, along with the full sequence of wild-type EXO5, can be found in Figure S1.

The N-terminal (residues 1–68) and C-terminal (residues 358–373) regions were not modeled due to their disordered nature. These regions have been shown in ref (12) not to contribute to EXO5 activity. All other missing residues (107–132), which include the conditionally folded α-helix enclosing the channel over the ssDNA, were modeled superimposing the 7LW9 structure with the complete EXO5 structure we obtained using ColabFold.^20,21^ In ColabFold, the AlphaFold2 model was used on the whole G172V EXO5 sequence, using the default MSA construction, no PDB templates, no relaxation of the structure, and 12 recycles through the folding trunk of the model. Extraneous ions present in the 7LW9 PDB were then removed and the single Mg^2+^ ion resolved in the 7LWA was then copied to the final PDB after alignment of the two protein structures.

**Figure 1:**
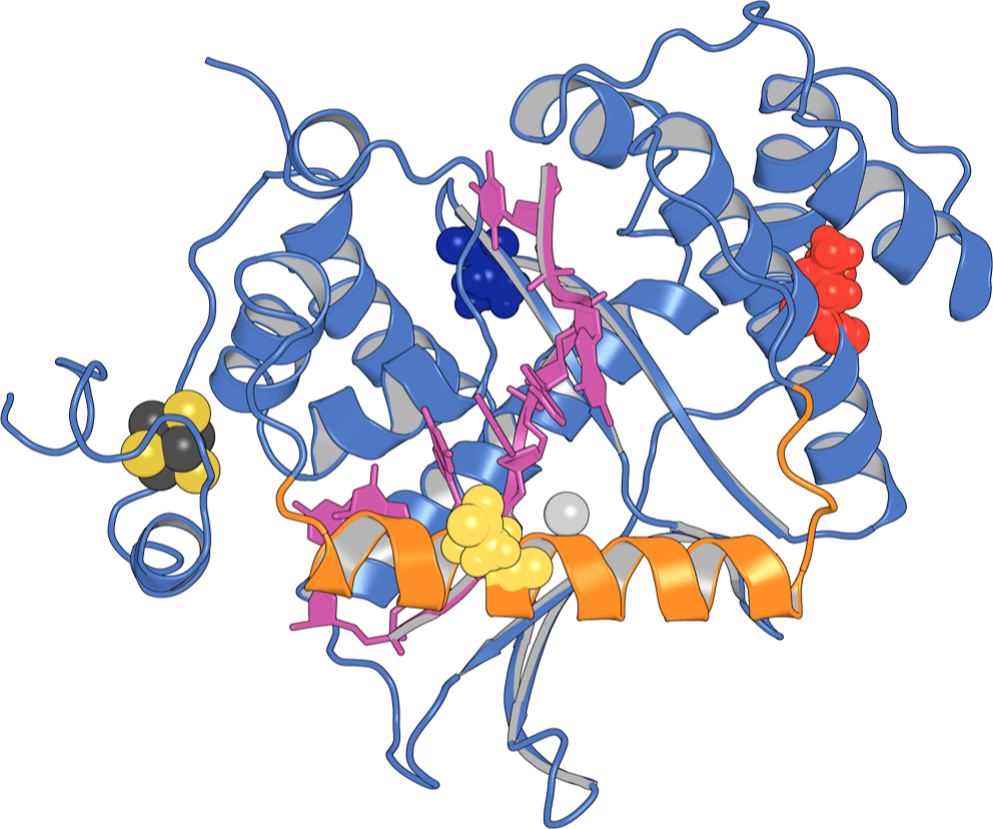
Illustration of the G172V EXO5 variant that was used as the starting structure of our simulations. In magenta the ssDNA, in light blue the protein structure taken from the 7LW9 PDB file, in orange the region extracted from the ColabFold prediction, which includes α4. The positions of the residues corresponding to the germline SNPs we have considered are marked with a sphere representation for the whole amino acid: blue for G172, red for L151, and yellow for D115. The catalytic Mg^2+^ ion is represented as a gray sphere, and the [4Fe–4S] iron–sulfur cluster with yellow and dark gray spheres for sulfur and iron atoms, respectively.

The resulting G172V EXO5 structure was then mutated to obtain the other EXO5 variants using PyMOL’s mutagenesis tool. The protonation of all structures was performed separately using PROPKA3,^22^ via the playmolecule Web server.^23^ A doubly protonated histidine (H117) was manually modified to have a single protonation state. The obtained EXO5 starting structure is shown in Figure 1.

The protein was described by the ff14SB Amber force field,^24^ in conjunction with the parmBSC1 force field for the ssDNA.^25^ The Li–Merz parameters were used for ionic species (potassium chloride and magnesium).^26^ The iron–sulfur cluster was modeled based on the DFT-optimized geometry detailed in ref (27), specifically in its reduced state A_2+_. Four covalent bonds between the Fe atoms of the iron–sulfur cluster and protein cysteines C92, C356, C359, and C365 were finally added using AmberTools23.^28^

#### Simulation Protocol

The EXO5 variants’ structures were used to create distinct simulation systems. Each system was solvated in water with a 15 Å buffer distance, and 187 potassium ions and 169 chloride ions were added to reach a 150 mM KCl concentration as well as ensuring the system’s overall charge neutrality.

MD simulations were carried out with Gromacs 2023.3.^29^ All systems underwent energy minimization by employing the steepest descent algorithm until a maximum force of 750 kJ mol^–1^ nm^–1^ was reached. This was followed by a heating phase of 50 ps, from 0 to 310 K. The solvent was thus thermalized in the canonical (*NVT*) ensemble at *T* = 310 K for 250 ps, employing the V-rescale thermostat^30^ (τ_*T*_ coupling constant of 0.2 ps) with an integration time step of 1 fs. Then, an equilibration step of the solvent in the isothermal–isobaric ensemble (*NPT*) at *P* = 1 bar was carried out for 1 ns, via the C-rescale barostat^31^ (τ_*P*_ = 2.0 ps, compressibility of 4.5 × 10^–5^ bar^–1^). During both stages, position restraints were applied to the magnesium ion as well as to all heavy atoms of the protein and DNA, using force constants of 1000 kJ mol^–1^ nm^–2^. A final unrestrained *NPT* equilibration was performed for 2 ns with a time step of 2 fs.

For all systems, a 4 μs-long trajectory was produced, after which the first 960 ns were cut. The frames at 960 ns were used to start (with randomized velocities) other 2 independent replicas of each system. Ultimately, 3 production replicates of 3 μs each (in total 9 μs per system) were obtained for each EXO5 variant.

MD equilibration and production stages in the *NPT* ensemble were carried out using an integration time step *t*_step_ of 2 fs, the v-rescale thermostat with τ_*T*_ = 0.2 ps, *T* = 310 K, and the C-rescale barostat with τ_*P*_ = 2 ps at 1 bar. For the solvent, including all ions present in the system and the solute, separated temperature couplings were used. Electrostatic interactions were treated using the PME method^32,33^ a PME order of 4. A cutoff of 1.1 nm was employed for both electrostatic and van der Waals interactions. Each bond involving hydrogen was constrained with the LINCS algorithm.^34,35^ Dispersion corrections for energy and pressure were applied by using the dispcorr = EnerPres GROMACS parameter, as recommended for simulations employing Amber force fields to account for long-range van der Waals interactions.

### MD Trajectories Analysis

The evolution of the EXO5 protein variants’ secondary structure elements, particularly in the region surrounding the α4 helix, was assessed using the MDAnalysis implementation of the PyDSSP algorithm.^36,37^ This is a simplified implementation of the original DSSP algorithm,^38^ where β-bulges are determined as loops instead of β-strands. Hydrogen positions were explicitly parsed from the trajectories instead of guessed.

MDAnalysis was also used to calculate the root mean square deviation (RMSD) and root mean square fluctuations (RMSF) over the trajectories with a sampling frequency of 200  ps for both RMSD and RMSF. The structures were aligned to the protein core, which excludes α4 because of its high mobility. The reference structure for RMSD was the PDB structure, while for the RMSF the average frame of the equilibrated trajectories was used. Different replicas were treated separately, averaging over the final results in the case of RMSF.

When an uncertainty on average values is reported, it represents the standard error of the mean, calculated based on the averages across replicas. The variance of the average estimate for each replica was derived using the moving block bootstrap method applied to all frames within that replica, unless otherwise specified.^39^ The block size was determined by estimating the autocorrelation time of the relevant time series as the time lag required for the autocorrelation function to reach the value *e*^–1^.

#### Protein Structure Network and Network Similarity Measure

In order to characterize the protein structure networks of our systems, we process the MD trajectories using PyInteraph 2.^40,41^ In this framework, the protein is described as a graph with residues as nodes and edges defined by noncovalent interactions, which in the case of PyInteraph 2 are characterized with user-defined geometric criteria corresponding to different chemical interactions. We defined the edges using atomic contacts between side chains, using the PyInteraph default residue and atomic interaction definitions for hydrogen bonds, salt bridges, and hydrophobic interactions.

The network was then generated from the equilibrated trajectories, excluding the first 960 ns of the first replica, with a frequency of 100 ps. The resulting adjacency matrices were weighted based on the occurrence of the interactions, i.e., the fraction of frames where the contact is detected divided by the total number of frames. The networks for each trajectory were then combined by taking, for each edge, the maximum occurrence value among the three types of interaction. A set of unweighted networks was also extracted from the weighted ones, using an occurrence cutoff of 20%, in line with previous literature.^40^

We then used the Jaccard similarity coefficient to evaluate the similarity across the different unweighted networks. This coefficient was calculated by dividing, among all pairs of trajectories, the number of common edges by the number of edges present in either one of the two networks.

#### Protein–DNA Interface Analysis

The interface between the EXO5 structures and the ssDNA ligand was examined by computing the atomic contact map between the two, defining the contacts as having a distance below 4 Å, with a smooth cutoff of up to 5 Å given by the function
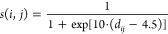


For mutated residues close to the DNA interface, we kept only atoms in common among all systems. The contact maps obtained were then clustered using the advanced density peaks clustering algorithm^42^ implemented in the DADApy package.^43^ Following previous literature,^44^ we used the Manhattan distance as the distance metric between the contact maps, after summing the contributions of atoms in the respective residues and rounding the contact number for each residue–nucleotide pair to the nearest integer.

#### Dimensionality Reduction and Clustering

With the aim of comparing the essential dynamics of EXO5 in the simulated trajectories, we performed principal component analysis (PCA) and computed the root mean square inner product (RMSIP) of the resulting essential subspaces.^45−47^ Different replicas were analyzed separately, allowing for an assessment of the convergence of the simulations along with the comparison between the different EXO5 protein variants.

PCA was performed at the α-carbon positions of the protein trajectories. Prior to performing the PCA, the trajectories were aligned via the alpha-carbons of the first equilibrated frame of G172V EXO5, excluding residues 107–131. A frame every 0.5 ns was analyzed, resulting in about 30,000 configurations per trajectory. The first 30 principal components of each trajectory were then used to compute the RMSIP. Both the PCA and the RMSIP calculation were performed using MDAnalysis.

The protein conformations were also clustered using the advanced density peaks clustering algorithm^42^ implemented in the DADApy package.^43^ Instead of using the 3D coordinates of the protein backbone, the ϕ and ψ backbone dihedrals were used as input, following previous research indicating that distance measures based on backbone dihedrals are more effective for clustering purposes.^48^ Protein dihedrals of the α4 region spanning residues 107–131 and the set of all other protein dihedrals were clustered separately, respectively, with a minimum uncertainty for the cluster separation of *Z* = 5 and *Z* = 3. This separation was chosen based on preliminary analyses that showed most of the structural diversity between different trajectories was driven by the residues 107–131. Clustering was performed on the concatenated EXO5 trajectories, with a total of 38,000 frames.

### Survival Analysis and Genomic Instability Analysis Using TCGA Data

Genotype and ancestry data of 8535 TCGA cancer patients were retrieved from ref (49) where unphased genotypes for exonic SNPs were computed using^50^ and ancestry information was retrieved by means of.^51^ Genotypes were then phased with SHAPEIT v2^52^ to infer haplotype structure using 1000 Genomes Project genotype data as reference panel. Importantly, since the ancestry composition of individuals in the 1000 Genomes Project closely mirrors the population structure of TCGA, this ensures robust and reliable haplotype reconstruction. *EXO5* haplotypes were then used to perform survival analysis using TCGA cancer survival data.^53^ Overall survival (OS) and progression-free interval (PFI) were considered in our analysis. Patients were stratified based on the presence of specific haplotypes. Kaplan–Meier survival curves were generated, and multivariate Cox proportional hazards regression models were applied, including ancestry and age at diagnosis as covariates. For PCa, a significance threshold of *p* < 0.05 was used. For the extended analysis across other cancer types, only those with more than 100 patients were included, and multiple hypothesis correction was applied using a *q*-value threshold of 0.05. Survival analyses were performed using the survival *R* package.^54^

Genomic instability analysis was conducted using data from.^55^ Specifically, weighted genome instability index values were obtained and analyzed for 310 patients with prostate or pancreatic cancer. Patients were stratified based on the presence of at least one allele carrying a specific *EXO5* haplotype. Individuals with significant aneuploidy—defined by an allele-specific aneuploidy score below 1.5 or above 2.5—were excluded.

## Results

### Haplotype Structure of *EXO5* Gene Across Human Populations

To characterize the haplotype structure of the *EXO5* gene across diverse human populations, we utilized genome-wide phased genotype data from approximately 2000 individuals obtained from the 1000 Genomes Project. We focused on identifying all missense SNPs and INDELs within the coding regions of all annotated *EXO5* gene transcripts with the aim of focusing on common germline variants that could potentially alter the functional properties of the translated protein. Notably, although ENSEMBL v111 reports multiple transcripts for the *EXO5* gene, the coding sequence remains uniformly conserved within the final exon across all transcripts. Indeed, all transcripts are annotated as encoding the same protein, Q9H790, which consists of 373 amino acids.

**Figure 2:**
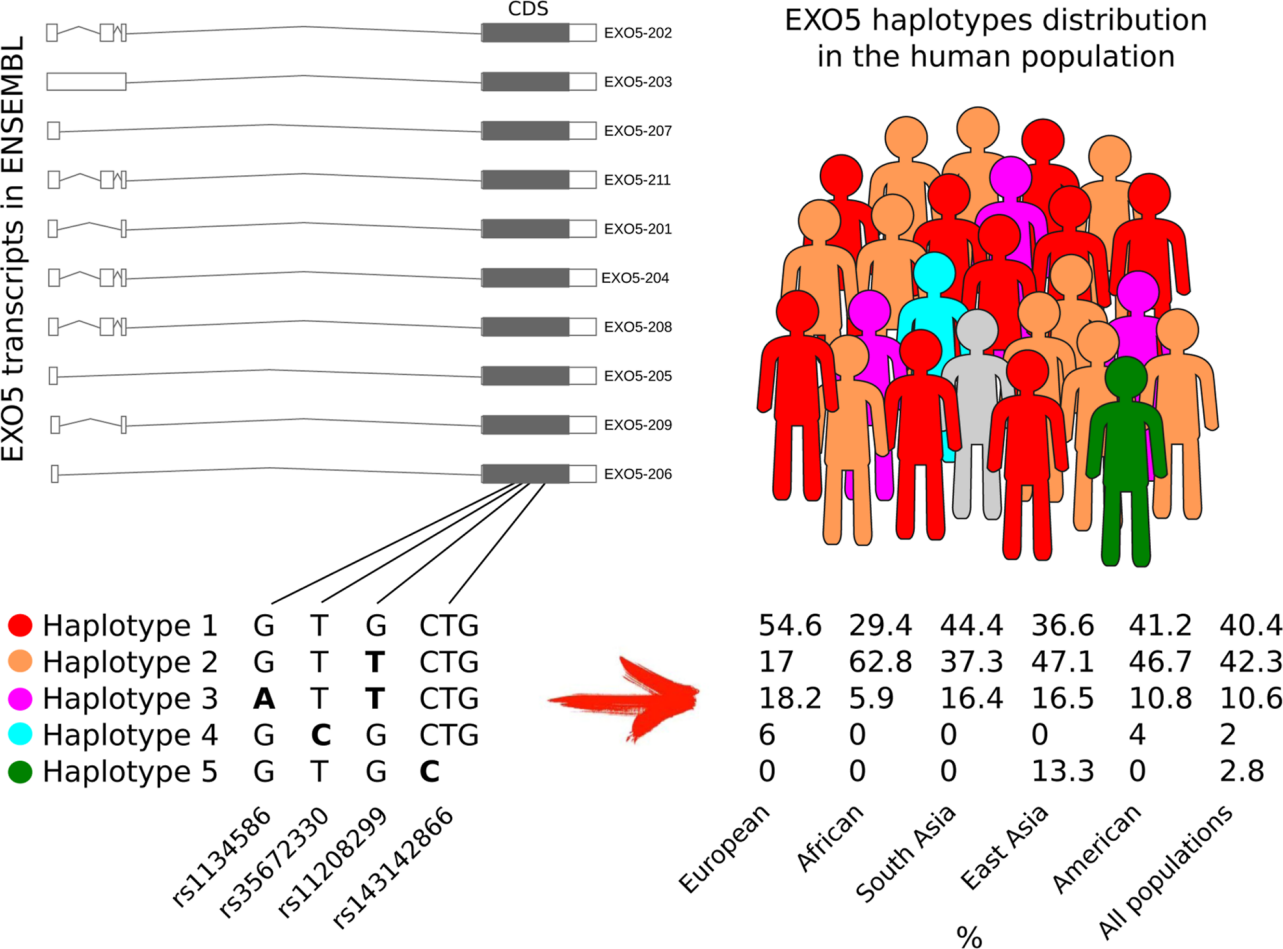
*EXO5* haplotype structure across major human populations. On the left, the annotated EXO5 transcripts are depicted, along with their shared coding sequence (CDS), highlighting the position of the coding SNPs that define the common *EXO5* haplotypes. On the right, the distribution of these *EXO5* haplotypes is shown across the global human population and across the major populations.

As shown in Figure 2, we identified five common haplotypes of the *EXO5* gene, each with a frequency exceeding 1% across all individuals analyzed (around 4000 haplotypes), collectively accounting for over 98% of the population. *Haplotype 1*, here also termed the wild-type (WT), corresponds to the reference human genome sequence (hg38) and was observed with a frequency of 40.4%. *Haplotype 2*, with a frequency of 42.3%, resulted as the most prevalent haplotype and was characterized by the presence of the alternative allele in SNP rs11208299. *Haplotype 3*, which carries the alternative alleles at both rs11208299 and rs1134586 SNPs, follows with a frequency of 10.6%. *Haplotype 4*, associated with the alternative allele at SNP rs35672330, was observed with a frequency of 2%, while *haplotype 5*, which includes the small deletion rs35672330, occurred with a frequency of 2.8%.

Interestingly, the distribution of *EXO5* haplotypes varied significantly among different populations. The WT haplotype was predominantly enriched in the European population, whereas *haplotype 2* was more frequently observed in African individuals. Despite its similarity to *haplotype 2*, *haplotype 3* exhibited a distribution pattern closer to that of the WT. Notably, *haplotype 4* was found only in Europeans and admixed Americans, while *haplotype 5* was exclusively observed in the East Asian population.

Overall, the haplotype structure of the *EXO5* gene was heterogeneous both across individuals and populations and predominantly consisting of SNPs’ patterns.

### Quantification of Common Human Haplotypes Impact on EXO5 Protein Structure and Function

Although the germline variants defining the common *EXO5* haplotypes we identified are annotated as benign in ClinVar,^56^ we reasoned that recent advances in deep learning approaches could help us screen for any potential, even subtle, functional impact these haplotypes might have on the EXO5 protein.

In particular, to quantify the impact of missense variant patterns on the structure and function of the EXO5 protein, we employed the ESM-1v PLM.^13^ This model is notable for capturing long-range patterns and features essential for protein function and stability, making it particularly effective in assessing the impact of alterations in amino acid sequences^14^ as also demonstrated in refs (57 and 58) using experimental and functional data.

We focused our analysis on *haplotypes 1* through 4, as these exhibit distinct SNP patterns and are represented across multiple major populations. First, we generated all EXO5 amino acid sequences incorporating the modifications caused by these SNPs. We then applied the ESM-1v model to calculate the posterior log-likelihood ratio (PLLR), a functional score that quantifies the impact of haplotype SNPs at the protein level by comparing the model’s log-likelihood of the SNP-altered amino acid sequence to that of the WT sequence.

Compared to the WT amino acid sequence of *haplotype 1*, the sequences for *haplotypes 2* and 3, which are characterized by the G172V and G172V + D115N amino acid changes, respectively, showed positive scores of 5.05 and 4.51. In contrast, the *haplotype 4* sequence, characterized by the L151P amino acid change, presented a negative score of −2.74. These PLLR scores suggest that the G172V and G172V + D115N variants of the EXO5 protein may be more favorable compared to the WT protein, while the L151P variant appears to be significantly unfavorable.

**Figure 3:**
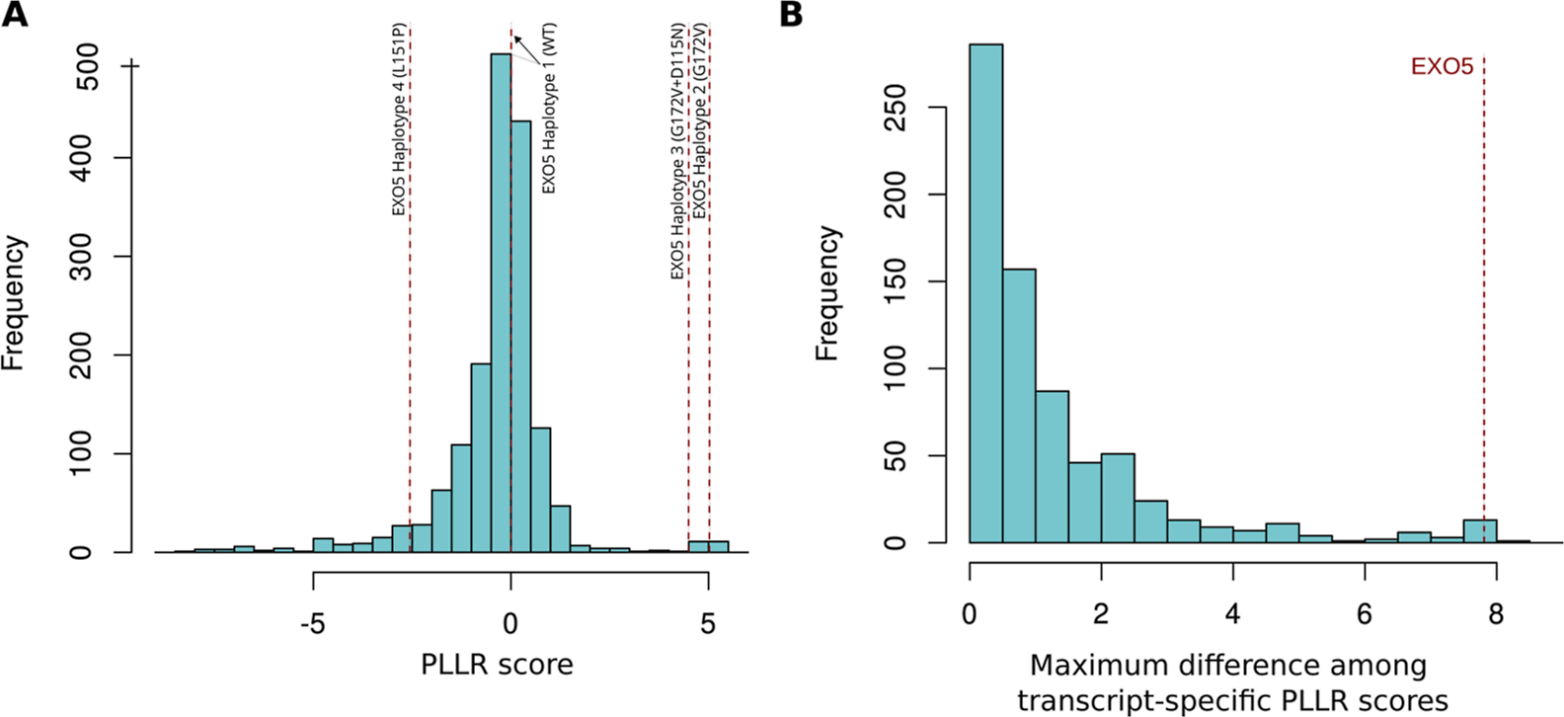
(A) Distribution of the PLLR scores calculated across all DDR and repair genes. (B) Distribution of the maximum difference of scores between variants inside each of the 174 genes with at least one common SNP.

To better assess the significance of the score variability observed for EXO5, we extended our analysis to a set of 251 genes associated with DDR and repair, identified from the literature.^16^ Specifically, we retrieved missense SNPs across these genes, determined the common haplotypes for all gene transcripts, and calculated the PLLR scores of these sequences (Table S1). The resulting score distribution, which includes the 174 genes observed to have multiple common haplotypes, is shown in Figure 3A. As shown, although the majority of PLLR scores clustered around zero (indicating haplotypes with no significant impact compared to the corresponding WT protein sequence), over 15% of haplotypes exhibited scores with an absolute value greater than 1. Furthermore, among the 721 transcripts with multiple haplotypes observed across all populations, 90 (12.5%) displayed a score variability (calculated as the maximum difference between transcript specific PLLR scores) greater than 1 (Figure 3B). Notably, the EXO5 score variability of 7.8 was situated at the upper tail of this distribution, second only to RAD51B, which showed a maximum variability of 8.5. Additionally, an analysis of cancer somatic mutational profiles from the TCGA data set (via cBioPortal) revealed that among the genes with haplotype variability greater than 2, several genes, including *EXO5*, *RAD51B*, *NUDT15*, *SWSAP1*, *POLM*, and *CLK2*- are recurrently altered in at least 2% of patients across various cancer types, based on data from approximately 11,000 TCGA patients.

Overall, these data strongly support the hypothesis that the various *EXO5* haplotypes, though common in the population, result in different EXO5 protein variants that may have distinct structures and functional properties, and could potentially interact with cancer somatic alterations.

### Mechanistic Effects of Common EXO5 Haplotypes on Protein Structure, Stability, and Dynamics

Considering the high variability of haplotype PLLR scores we observed for EXO5 protein variants and the availability of the resolved structure for G172V EXO5 in the PDB (7LW9), we investigated mechanistically the effects of different *EXO5* haplotypes using MD simulations. Starting from the available G172V EXO5 structure, we generated all of the EXO5 protein variants corresponding to SNP-based haplotypes. The initial structure, corresponding to *haplotype 2*, was thus reverted to the wild-type (*haplotype 1*) by mutating V172 to G172. From the WT EXO5 structure, L151P EXO5 was then obtained (*haplotype 4*), while adding the D115N-substitution to the G172V EXO5 structure resulted in the protein associated with it (*haplotype 3*).

We then performed MD simulations of all systems in the presence of an ssDNA, in order to better characterize the native structure and dynamics of the active state of the nuclease. Such a computational approach is well suited to catch the conformational dynamics of the DNA strand.^59^ Its binding region, being directly influenced by residue substitutions, is especially sensitive to haplotypes. Protein’s activity, in fact, can be allosterically modulated by the binding with biological molecules,^60−62^ and be affected by mutations.^63^

**Figure 4:**
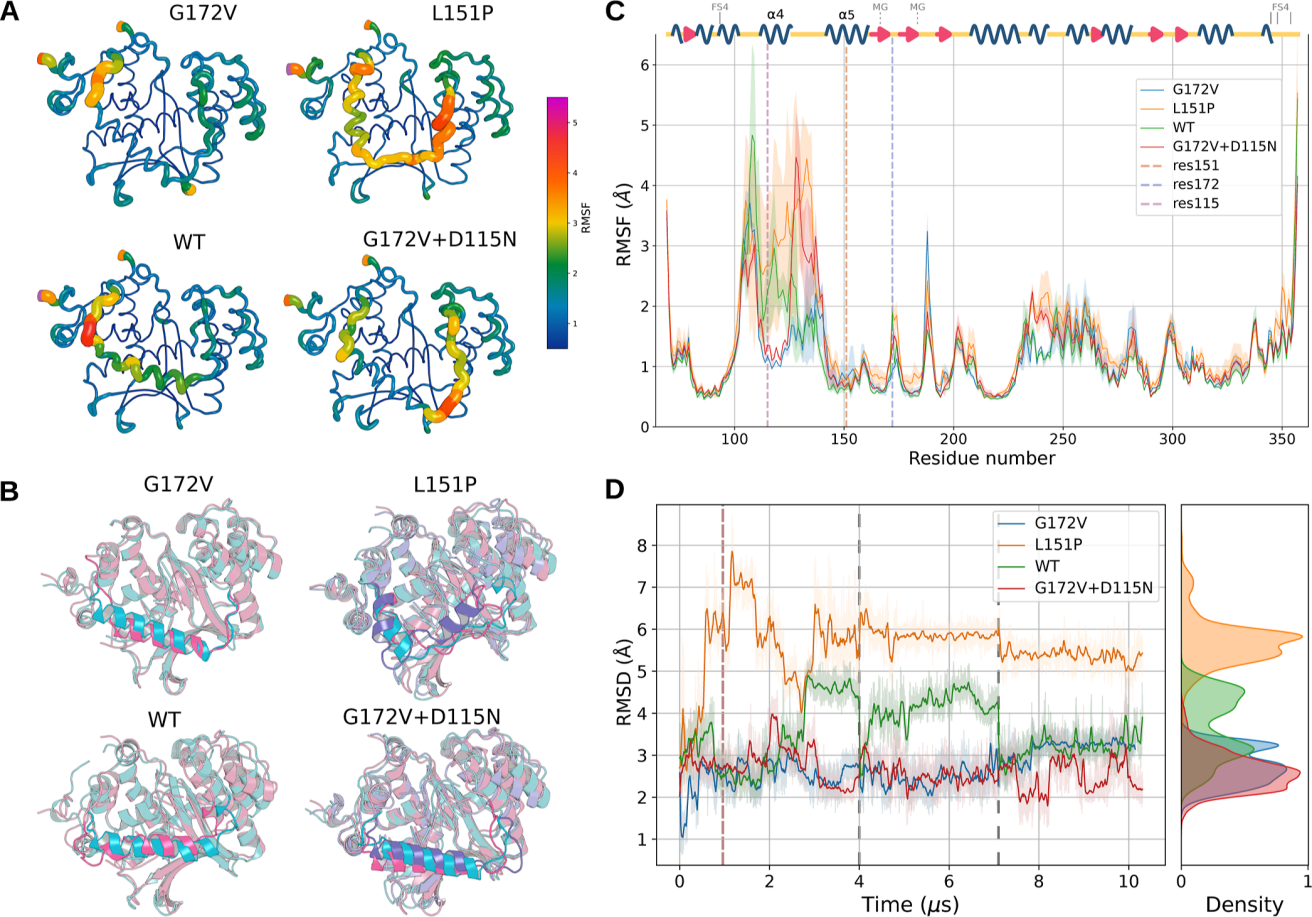
(A) Average conformation of EXO5 structures across the equilibrated trajectories. The size and color of the local representation are given by the RMSF for each residue. (B) Representative EXO5 structures with the ssDNA ligand. Each color represents one cluster center as found by clustering the backbone dihedrals of residues 106–133 obtained from the equilibrated trajectory. Only clusters containing at least 10% of the total frames are shown. Residues 106–133, which include the α4 helix, are highlighted in front of the protein structures. Full clustering results are reported in Figure S5. (C) Average RMSF of EXO5 calculated separately for all replicas, with confidence intervals calculated as the standard error of the mean. On top of the RMSF plot, the secondary structure of G172V EXO5 is shown, as computed on the whole MD trajectory and then filtered to show as structured residues only the ones with the same assigned type for more than 70% of the frames. The sequence positions of residues coordinating the magnesium ion or forming the coordinate bonds with the iron–sulfur cluster are also pointed out as dashed lines. (D) RMSD of the α4 region (residues 107–131) across the whole trajectory. Lighter shades are the raw data, calculated every 0.5 ns, while the darker lines are a moving average with a 40 ns uniform window. The dashed red line indicates the chosen equilibration cutoff, while the dashed gray lines separate different replicas.

None of the haplotypes produced a global destabilization of the protein tertiary structure, but significant differences, consistent among different replicas, were apparent in the structure and dynamics of the region spanning residues 106 to 133, comprising the α4 helix and the two disordered arms connecting it to the core of the protein (Figure 4A).

The α4 helix, resolved in ref (12) for EXO5 with no ligands, was absent in the DNA-bound G172V EXO5 structure, suggesting it may be a conditionally folded region or exhibit higher mobility in the presence of ssDNA. AlphaFold 2 (for G172V EXO5) predicts this region to form a 19-residue-long helix spanning residues 109 to 127. This helix, along with the disordered linkers, is proposed to form a channel for ssDNA. In contrast to the DNA-free structure of 7LW7, the position and length of helix α4 in the predicted structure differ in order to accommodate the ssDNA.

#### Alpha Helix 4 Undergoes Big Conformational Changes in L151P and WT EXO5

Our simulations showed an overall stability of the α4 helix conformation for the G172V EXO5 variant, with an average 5.5 Å RMSD with respect to the starting EXO5 structure (Figure S2) and a 2.5 Å RMSD when aligning the 106–133 region alone (Figure 4D). This discrepancy is the result of a component of the rigid motion of the α4 region. Indeed, in G172V EXO5 simulations, the disordered linker corresponding to residues 128–138 relaxed toward α5, pulling toward this region and thus toward the DNA channel the N-terminal part of α4 as well. Despite this displacement, which is common to all of the simulated EXO5 variants, the local conformation predicted by Alphafold for G172V EXO5 is overall confirmed. This stability is also supported by RSMF values (Figure 4C) and by an analysis of the region’s secondary structure performed with PyDSSP (Figure S4), which shows an average difference of 20% between the secondary structure assignment during the (equilibrated) simulation and in the starting PDB structure, mostly due to the formation of transient secondary structures within the linkers adjacent to α4 (Figure S3B).

Notably, the addition of the D115N SNP does not disrupt the α helix 4 conformation (Figure 4A), with an average RMSD around 2.5 Å and a secondary structure compatible with G172V EXO5 (Figures 4B and S3), but results in higher RSMF values in residues 124–135 (Figure 4C), the linker adjacent to α5 and α7, suggesting possible changes in the allosteric interactions between α4 and other EXO5 regions.

When focusing on the L151P EXO5 protein variant, α4 and the surrounding linkers were observed undergoing substantial structural rearrangements, higher RMSF values, and changes in the secondary structure (Figure 4). This conformational change is clearly visible in Figure 4A,B, where the average and the most common conformations of L151P EXO5 show a substantial loss of the original α4 helix structure, replaced by a disordered region that spans the residues between W116 and W137. The only persisting stable α-helical structure is formed between residues P108 and T115, partially replacing the short disordered linker in the starting structure. This reorganization is reflected in a lower fraction of ordered residues in the α4 region of L151P EXO5 (ordered fraction of 0.422 ± 0.033) compared to WT EXO5 (ordered fraction of 0.534 ± 0.046) and to G172V EXO5 (0.524 ± 0.043). The fraction of secondary structure assignment changes compared to the starting structure, as shown in Figure S4, as well as the RMSD in Figure 4D, highlights the strong deviation of the α4 region in L151P EXO5.

Finally, when analyzing the WT EXO5 variant, we observed a high variance between replicas in the RMSD and secondary structure metrics of the α4 region. For the first 2.5 μs of the first replica, the dynamics are similar to those of the G172V EXO5. At 2.5 μs, as well as shortly after the beginning of the second replica, the α helix exhibits a loss of stability corresponding to higher RMSD values and changes in the secondary structure (Figure 4C,D). However, even during those time spans, we did not observe such a drastic conformational change as in L151P EXO5. As indicated by the RMSF values (Figure 4C), and opposed to what happens in L151P EXO5, it is the short disordered linker composed by residues 103–109 that manifests higher RMSF values, with the long linker on the opposite side maintaining RMSF values below 2 Å, compatible with the ones of the G172V EXO5. The integrity of the α4 helix exhibits instability, falling between the behaviors observed in EXO5 L151P and G172V variants.

Overall, L151P EXO5 exhibits the highest mobility and conformational variety in the α4 region, resulting in unique, highly disordered, conformations, as evidenced in Figure 4B. On the other hand, the two EXO5 variants with the G172V change present the greatest similarity to the predicted initial conformation and to each other. WT EXO5 shows intermediate behavior, adopting unique conformations with less variability and maintaining a secondary structure that more closely resembles the one predicted by AlphaFold, compared to L151P EXO5.

These results can also be observed when clustering the dihedral angles in the region (Figure S5). Notably, apart from a minor fraction of frames where G172V and G172V + D115N EXO5 variants share the same cluster, each remaining cluster is predominantly associated with a single EXO5 variant. In particular, the L151P EXO5 variant displays a greater diversity of clusters with many clusters containing frames unique to only one of the three replicas. A similar pattern emerges when clustering the contact maps between EXO5 protein variants and the ssDNA molecule (Figure S8). The contact map clusters for different EXO5 variants are well-separated, mirroring the distinctions observed in the α4 conformations. L151P EXO5 shows the greatest variability and the largest deviation from other systems, while WT EXO5 exhibits intermediate behavior. Notably, the most frequent WT cluster, formed primarily by frames from the first and third replicas, appears more similar to those of G172V and G172V + D115N than to L151P.

Remarkably, the stability of α4, part of the catalytic region of EXO5, correlates with the predicted fitness scores produced by the ESM PLM analysis, even if none of the mutated residues that influence the scores are structurally close to α4, except for N115.

#### Structural and Dynamical Consequences of the L151P-Substitution Propagate from a Local Kink in Helix α5

**Figure 5:**
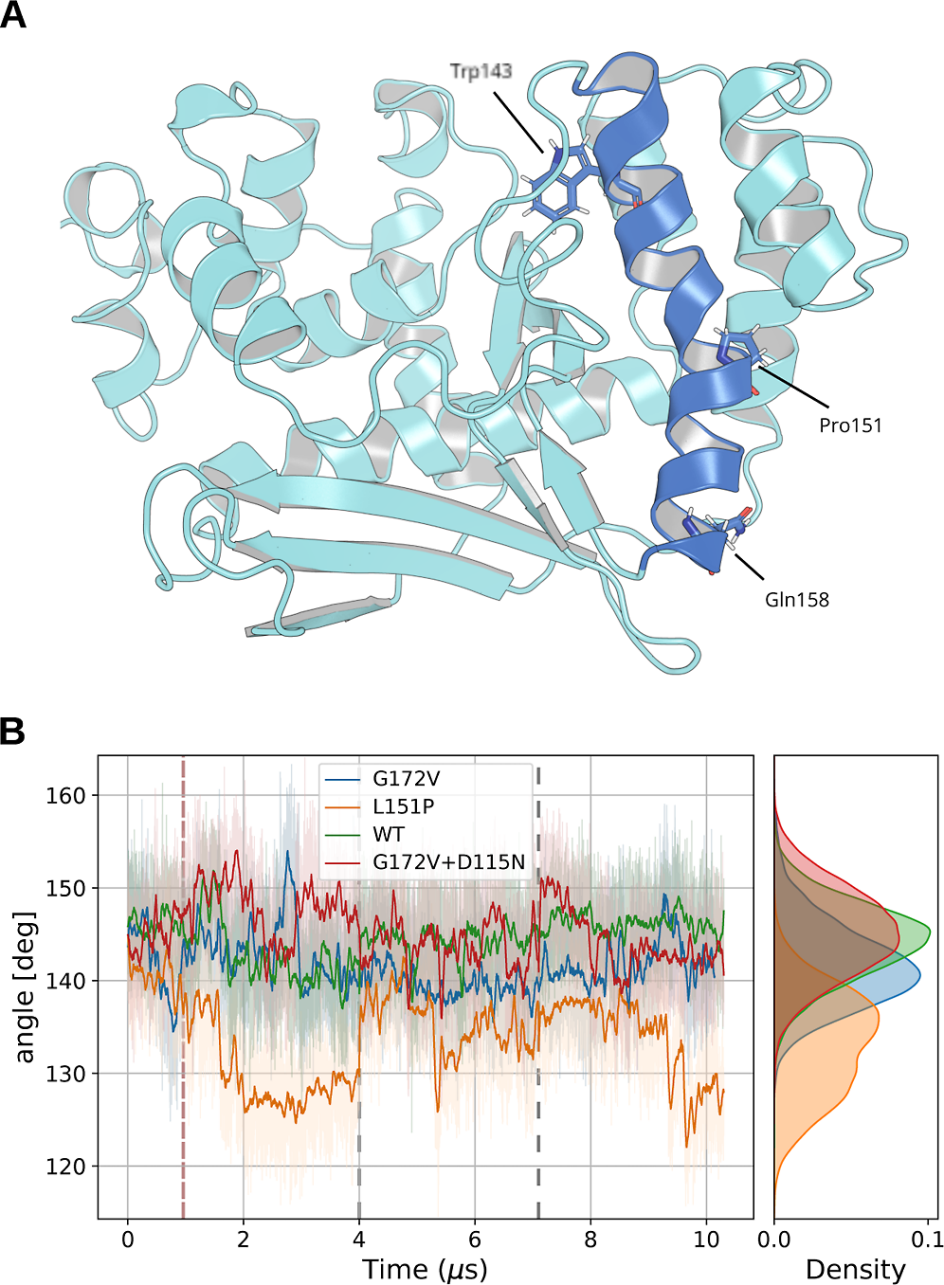
Illustration of a representative structure of L151P EXO5. α5 is highlighted in blue, and the P151 residue is highlighted, along with the residues used to compute the angle formed by the α-helix. The trajectory frame was chosen as the center of the biggest cluster along all replica of the L151P EXO5 MD trajectory (A). Angle formed by W143, P151, and Q158 (B). Lighter shades are the raw data, calculated every 0.5 ns, while the darker lines are a moving average with a 40 ns uniform window. The dashed red line indicates the chosen equilibration cutoff, while the dashed gray lines separate different replicas.

It is known that proline substitutions limit the flexibility of their peptide bonds, disrupt the local hydrogen bond network, and introduce a kink when found in the middle of alpha helices. Indeed, the most direct effect of the L151P-substitution in EXO5 is the formation of a kink in α5, as revealed by the changes in the angle formed by its extremes, with L151 as its vertex (Figure 5A). Although the kink formation occurs in a time scale of microseconds, possibly indicating a suboptimal sampling with our simulation time, the angle sharply decreases in all L151P EXO5 replicas, reaching values below 120° (average 133.0° ± 3.3°), compared to an average of 144.3° ± 1.8° in WT EXO5 (Figure 5B).

This kink in α5 likely contributes to the destabilization of α4, a hypothesis further supported by the increased RMSF observed in the L151P EXO5 variant (Figure 4C), particularly within the linker region connecting these two alpha-helices.

This strongly suggests that the stability of α4 is likely compromised by L151P-substitution through an allosteric pathway that propagates across, or through, the linker between these regions.

**Figure 6:**
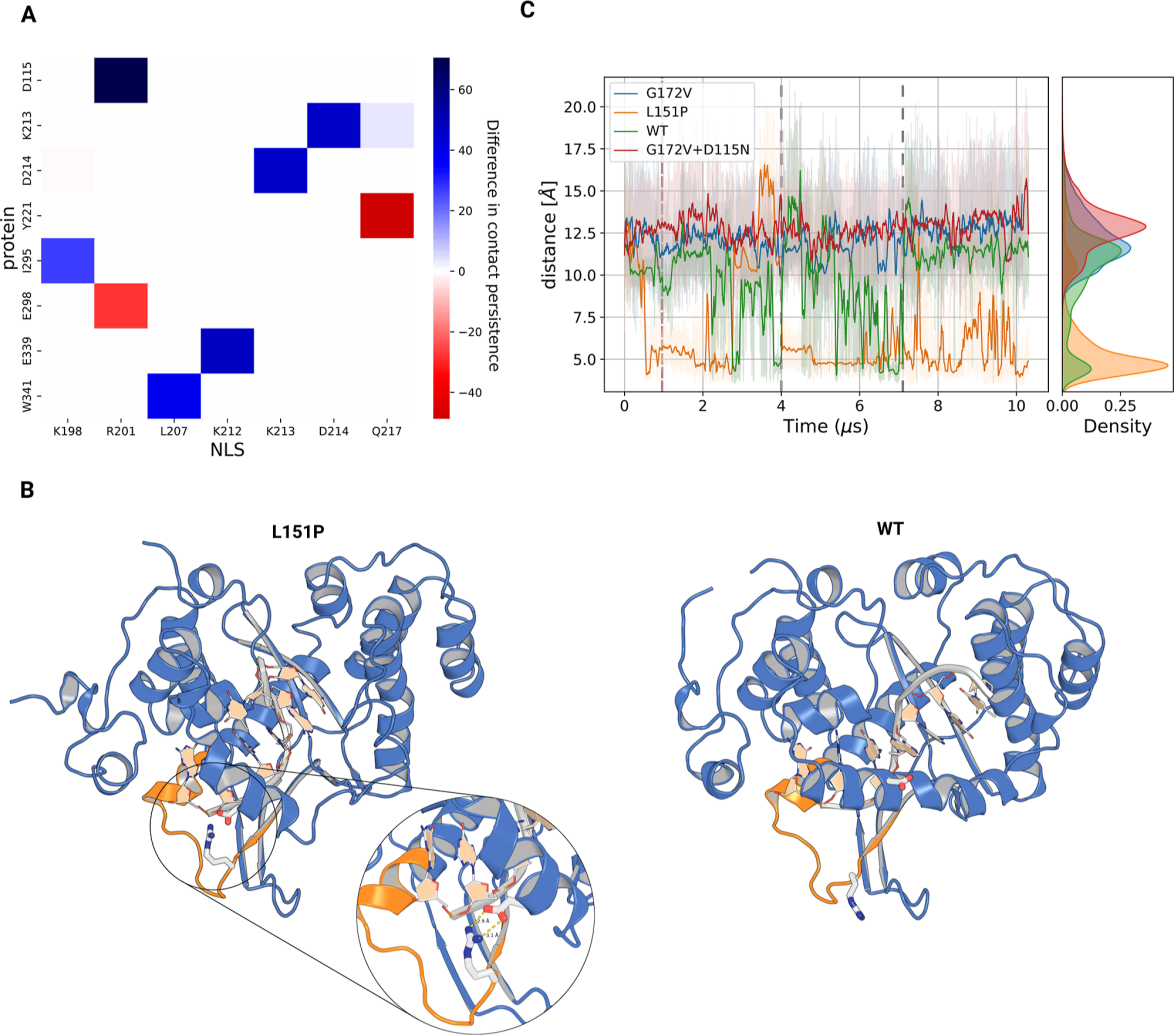
(A) Difference in the occurrence of contacts between the nuclear localization sequence and the rest of the protein, in the MD trajectory of L151P EXO5 vs the trajectory of G172V EXO5; (B) illustration of the NLS (orange), with R201 and D115 represented with blue and white sticks and red and white sticks, respectively. The wild-type system is shown below for comparison. The structures shown are the representative frames of the respective system’s trajectories, as found with clustering, by identifying the frame with the highest logarithmic density inside the largest cluster. (C) Distance between R201 (zeta carbon) and D115 (gamma carbon) over time. Lighter shades are the raw data, calculated every 0.5 ns, while the darker lines are a moving average with a 40 ns uniform window. The dashed red line indicates the chosen equilibration cutoff, while the dashed gray lines separate different replicas.

The conformational changes in the region comprising α4 and its surrounding disordered loops are also found to allosterically propagate the effects of the L151P-substitution on the opposite side of the protein. This region is particularly interesting because it contains a sequence motif (KTRRRPMLPLEAQKKK, residues 198–213), which is classified with a high confidence by INSP^64^ as a nuclear localization signal (NLS). Especially relevant are the arginine and lysine triplets, which are known targets of the Importin class of transport proteins. The network of interactions made with PyInteraph was analyzed (Figures 6A and S6), which shows that in L151P EXO5, the middle arginine of the polybasic motif (R201) forms a stable salt bridge with D115, an aspartate that forms a transient α helix with the surrounding residues only in L151P EXO5 (Figure 6B). In 70.5% of the equilibrated frames (Figure S6), the charged side chains of R201 and D115 are less than 4.5 Å distant. In Figure 6C, it can be seen that this interaction occurs in all simulation replicas, while for WT EXO5, it is a transient interaction, with R201 and D115 isolated for most of the 9 μs trajectories. This interaction is completely absent in G172V and G172V + D115N EXO5 (see also Figure S6).

Additionally, on the lysine triplet, K212 of L151P EXO5 forms a salt bridge with E339 (63.0% of occurrence) and K213 with D214, part of the same NLS motif. These contacts are only transient (14%–21% occurrence for K212-E339) in all other EXO5 simulations (see Figure S6). These newly formed contacts do not cause a large, observable conformational change in the NLS backbone, but they seem to result in a reduced mobility of the disordered loop instead. This is indicated by a much longer tail in the autocorrelation function of the NLS RMSD (Figure S7B), which corresponds to an estimated autocorrelation time 1 order of magnitude higher compared to the autocorrelation time for all replicas of G172V, WT, and G172V + D115N EXO5.

Not only the RRR and KKK motifs recognition by an importin could be hindered by a high affinity to D115 and E339 in L151P EXO5, but if this recognition is dependent on a specific conformation of the loop, the higher autocorrelation time of the NLS loop indicates that a lower loop mobility could considerably slow down the process.

#### Global Structural and Dynamic Differences Among EXO5 Variants

The similarity analysis of the entire EXO5 structure reveals a pattern consistent with that observed in the α4 region.

**Figure 7:**
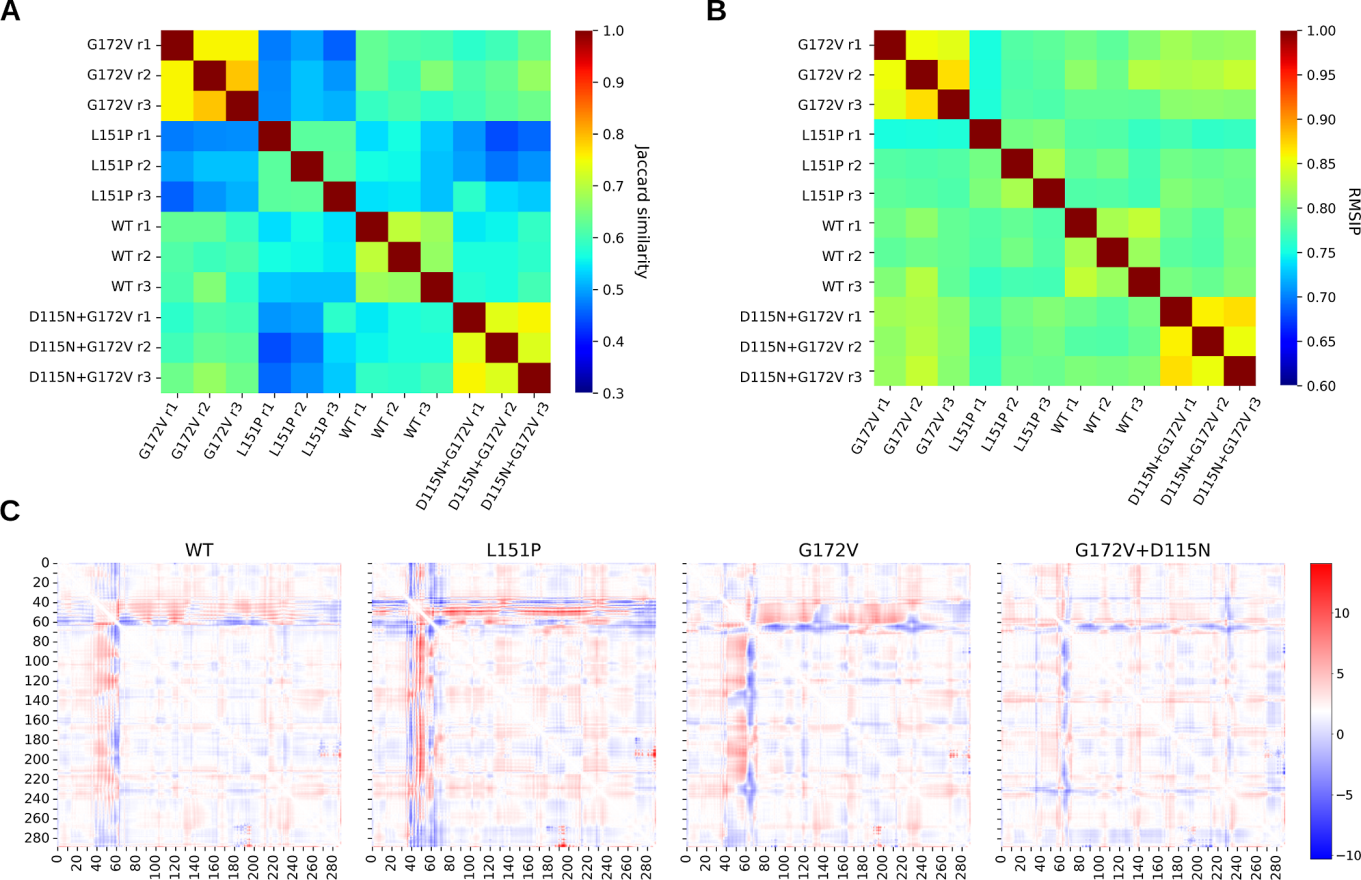
Heatmap of the Jaccard similarity between all unweighted protein structure networks derived from the Pyinteraph analysis, with a 20% cutoff of the occurrence of interactions (A). RMSIP of the essential subspace constituted by the first 30 principal components of each of the 3 MD simulation replicas per each EXO5 structure (B). Average change in distance between pairs of amino acids in the equilibrated trajectories, compared to the starting structures. In red are protein residues that on average across all replica tend to get farther away from each other; in blue are residues that on average get closer to each other. Note that residue indices are shifted by −68 compared to the original EXO5, and here they represent the residue index in the simulated structures (C).

The unweighted PSNs derived from the equilibrated trajectories of the G172V and G172V + D115N EXO5 variants show the highest similarity, as reflected in their Jaccard scores (Figure 7A). WT EXO5 also has comparable similarity to G172V EXO5, but slightly lower, compared to G172V + D115N EXO5. L151P is instead an outlier, with an average Jaccard similarity of 0.5 compared to that of G172V EXO5 and 0.55 compared to that of WT EXO5. While the semidisordered region in WT EXO5 is more similar to L151P EXO5 than to other variants, the overall interaction network of WT EXO5 aligns more closely with the G172V variants, indicating that its residue interaction network is less affected by the instability of α4. Both G172V and G172V + D115N EXO5 exhibit good convergence, with a high Jaccard similarity between replicas, whereas WT EXO5 and L151P EXO5 both display higher diversity, especially in the case of L151P EXO5.

Furthermore, the RMSIP of the essential subspaces (Figure 7B) shows that G172V and G172V + D115N EXO5, followed by WT EXO5, have the highest similarity in their essential dynamics, highlighting a strong consistency in conformational sampling across different replicas of these variants. In contrast, the RMSIP for L151P EXO5, when compared with other EXO5 variants, is similar to the RMSIP observed within L151P replicas, suggesting that the dynamic differences characteristic of L151P simulations are not readily apparent. The RMSIP values between WT and L151P EXO5 simulations are compatible with the ones between L151P EXO5 and the stable forms of EXO5, suggesting that the essential dynamics captured by principal components does not capture any meaningful similarities between the two forms of EXO5, which exhibit the strongest instabilities in the α4 region.

Figure 7C displays the average pairwise displacement of the α-carbons from the initial distance map. There is a consistency in the movement of the α4 helix away from the core of the protein in all EXO5 forms, as well as an approach of the linker between α4 and α5 toward α5. L151P, however, shows again a unique behavior, with residues 106–113 that, on average, tend to lay closer to the rest of the protein. α4 itself, on the contrary, moves away from the protein core and thus from DNA in a more pronounced manner with respect to the other EXO5 forms, allowed by its greater degree of disorder.

Overall, when focusing on local interactions, overall conformational changes, and essential dynamics, all metrics considered show distinct patterns for each EXO5 protein variant, with L151P EXO5 exhibiting the most divergent behavior.

### Impact of the L151P EXO5 Variant on Cancer Patients

Given the significant structural impact of *haplotype 4* on the EXO5 protein and the previous association of SNP rs35672330 with PCa risk characterized in ref (11), we investigated the distribution of *haplotype 4* in both healthy individuals and cancer patients, utilizing data from the 1000 Genomes Project and TCGA.

Utilizing TCGA genotype and ancestry data obtained from ref (49) via refs (51 and 65), we reconstructed the *EXO5* haplotypes for 8535 cancer patients spanning 24 cancer types and representing the five major population groups.

No cancer- or population-specific enrichment of *haplotype 4* was observed. Notably, focusing on European individuals, representing the majority of individuals in TCGA data, the frequency of *haplotype 4* was 5.93% in 1000 Genomes Project individuals and 6.18% in TCGA PCa data set, suggesting that, contrary to ref (11), SNP rs35672330 may not play a specific role in PCa predisposition.

**Figure 8:**
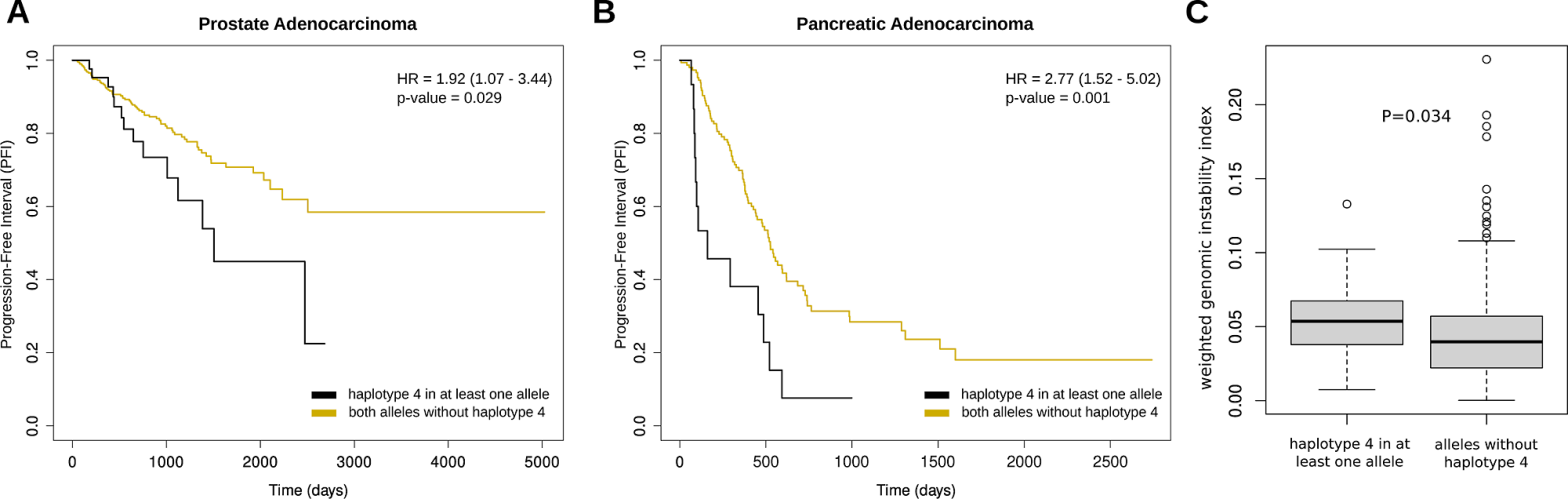
(A) PFI of patients with PCa stratified by the presence of *haplotype 4* in at least one allele; (B) PFI of patients with pancreatic cancer stratified by presence of *haplotype 4* in at least one allele. (C) Weighted genomic instability index values in patients with prostate and pancreatic cancer stratified by presence of *haplotype 4* in at least one allele. Statistic was performed using two-tailed Wilcoxon test statistics.

We hence hypothesized that the L151P EXO5 variant might influence PCa progression and/or aggressiveness. To explore this, we obtained TGCA OS and PFI data from ref (53) and stratified TCGA PCa patients based on the presence or absence of *haplotype 4*. We then conducted a multivariate Cox proportional hazards regression analysis, adjusting for age at diagnosis and ancestry. Notably, patients carrying *haplotype 4* on at least one allele exhibited significantly (*p*-value < 0.05) shorter progression-free survival (Figure 8A).

Intrigued by these findings, we expanded our analysis to include 17 additional cancer types from the TCGA data set, selecting for the ones comprising more than 100 patients. After correcting for multiple hypothesis, we identified pancreatic adenocarcinoma (*q*-value < 0.05) as another cancer type where patients carrying *haplotype 4* on at least one allele show significantly shorter progression-free survival (Figure 8B).

Given the hypothesis that worse progression in these patients could be driven by increased genomic instability, we retrieved genomic instability estimates from ref (55) and analyzed their association with the presence of *haplotype 4*. As illustrated in Figure 8C, patients carrying *haplotype 4* in at least one allele exhibited a statistically significant increase in the level of genomic instability.

Notably, repeating these analyses while focusing on *haplotypes 1*, 2, or 3 revealed no association with survival outcomes or genomic instability.

Overall, these findings support the hypothesis that the L151P EXO5 protein variant may contribute to earlier cancer progression across different cancer types by modulating genomic instability.

## Discussion

The study of common human genetic variants offers valuable insights into the biological mechanisms underlying complex traits and diseases. SNPs, the most prevalent type of DNA polymorphism, have been shown to influence cancer susceptibility and play a critical role in shaping treatment responses and clinical outcomes, particularly when located in genes encoding proteins involved in DDR and repair.^4,5^

In this study, we focused on EXO5, a single-stranded DNA exonuclease involved in DNA repair and previously associated with cancer susceptibility.^11,66^ Through an integrated approach that combines large-scale genomic data analysis, protein language models, and MD simulations, we investigated the impact of common human *EXO5* gene haplotypes on the structural and dynamic properties of the EXO5 protein. Additionally, we explored their potential role in cancer progression using data from comprehensive genomic data sets.

Our results demonstrate a strong correlation between the fitness predicted by protein language models and the maintenance of the structural integrity of the α4 helix and its adjacent linkers in EXO5. The importance of this protein region was demonstrated in ref (12), where it was found that mutations of conserved residues in the α4 helix can nearly abolish EXO5′s activity and reduce its binding affinity to ssDNA. We find that structural changes to this region are linked to changes in the protein-ssDNA interface, possibly explaining differences in DNA recognition and nuclease activity. The ESM-1v model correctly identifies the proline substitution L151P in α5 as causing structural instability, which aligns with experimental results obtained in ref (11), where the substitution was observed to cause a loss of EXO5 nuclease activity and of nuclear localization. Interestingly, it also identifies the G172V variant of the EXO5 protein as having a higher fitness than the WT EXO5, an insight that was not immediately explainable from structural considerations alone but was suggested by our MD simulations to be also related to the stability of helix α4.

While our MD simulations revealed consistent differences in the α4 behavior across systems and replicas, we remark that modeling partially disordered protein regions presents inherent challenges. The Amber ff14sb force field, combined with the TIP3P water model, may introduce some bias in helix propensity predictions.^67^ Furthermore, we found that the loss of stability resulted in a significant extension of the conformational space in this region, requiring long simulation time scales for appropriate sampling. For this reason, enhanced sampling schemes such as Hamiltonian replica exchange methods^68^ or Gaussian Accelerated MD^69^ might reveal alternative conformational states, potentially further away from the initial structure, that are difficult to capture with conventional MD simulations, further elucidating the impact of the mutations on EXO5 function. However, the robust patterns we observed across different systems suggest that our key findings regarding the structural and dynamic properties of the EXO5 variants are reliable. These results align well with both our PLM predictions and previous experimental observations, providing a multifaceted view of EXO5 dynamics.

In all systems, the α4 region maintains high mobility, but its conformational ensemble and its contacts with the rest of the protein appear to be unique to each EXO5 protein variant. The effects of amino acid substitutions are often nonlocal and allosteric in nature, and while in the case of L151P EXO5 we discovered a possible pathway by which the local disruption in α5 propagates, in the case of G172V, it is unclear from our simulations how the SNP related to a distant loop affects the behavior of α4. This highlights the complexity of the protein dynamics–function relationship and the importance of considering long-range allosteric effects, even if a substitution does not cause detectable local structural changes.

To understand the biological implications of these structural findings, we focused particularly on the L151P substitution. While it was previously experimentally observed in literature,^11^ our study provides additional mechanistic insights into its effects on EXO5 dynamics and the potential impact it has on EXO5 nuclear localization. The formation of new stable contacts involving the NLS region in the L151P EXO5 protein variant suggests a possible mechanism for a sharp decrease in nuclear transport binding affinity, which could result in a mostly nonfunctional EXO5, strongly supporting the findings from.^11^

Although it remains unclear whether this fully inactivates EXO5 in vivo, the analysis of a large cohort of cancer patients from the TCGA project shows that cancer patients carrying the rs35672330 SNP exhibit increased genomic instability and worse prognosis across multiple cancer types. These results, together with the findings by ref (12), which link EXO5 depletion to increased sister chromatid exchanges—a typical indicator of DNA repair defects or excessive DNA damage—support the hypothesis that the L151P protein variant, by significantly impairing EXO5 function, may exacerbate genomic instability, accelerating disease progression.

In summary, while providing important mechanistic insights on the germline determinants of EXO5 protein structure and dynamics and their possible role in cancer progression, our study also demonstrates the potential of combining large scale genomics data with deep learning variant effect prediction tools and MD simulations to explore the impact of common SNPs on protein structure, dynamics, and function.

This study primarily focused on the effects of SNPs, leaving *haplotype 5* aside. The latter, being characterized by the presence of an INDEL, introduces a premature stop codon. Hence, as a next step, we plan to integrate the current approach with experimental work to further explore the potential functional consequences of EXO5 germline determinants. Additionally, given that our extended PLM analysis identified other potential candidates, such as *RAD51B*, a more comprehensive exploration encompassing a full spectrum of genes involved in DDR and DNA repair^70^ could be considered.

From a technical perspective, sequence-only protein language models such as ESM-1v offer distinct advantages for our analysis. While structure-aware methods such as inverse folding^71^ and structure-augmented PLMs^72,73^ may achieve higher accuracy on average for structured proteins,^57^ they might show a systematic accuracy bias toward structured regions, as well as requiring input structures that often require a partial or complete computational determination, possibly introducing additional biases. Fitness prediction using PLMs likelihoods and sequences as the only inputs offers advantages in terms of speed and flexibility compared to many other methods, and while in this work, we computed the pseudolog-likelihood of sequences as the sum of log-likelihoods of amino acids, normalization of the PLLR on the sequence length constitutes a natural extension of the scoring method. This allows for the comparison between sequences of different lengths, as in the case of different isoforms or in the presence of indels, thus enabling the study of complete genetic landscapes. Future work could nonetheless explore the use of other models with different training objectives, different scoring methods, or supervised fine-tuning of experimental variant data. These approaches might allow us to distinguish the effects of genetic variants on structural stability and other fitness components.

To conclude, our methodology provides a novel and valuable computational framework to investigate the local interaction of multiple germline variants, which complemented with orthogonal approaches,^74,75^ could enhance our ability to explore the interaction landscape of common genetic variants and somatic phenotypes and signatures^76−80^ that underpin cancer risk and progression.

## Conclusions

Our study provides mechanistic insights into the impact of common *EXO5* haplotypes on protein structure, dynamics, and cancer susceptibility. By integrating large-scale genomic data, protein language models, and MD simulations, we identified key structural determinants of the EXO5 function, particularly within the α4 and α5 helices. The predicted destabilizing effect of the L151P variant is consistent with prior experimental findings, supporting its functional relevance. Furthermore, our TCGA analysis indicates that L151P may contribute to increased genomic instability and poorer cancer prognosis.

Beyond EXO5, our findings underscore the potential of combining deep-learning-based variant effect prediction with molecular simulations to systematically investigate the functional consequences of common SNPs. Experimental validation will be critical to further elucidate the biological significance of these observations.

From a methodological perspective, our study highlights the advantages of sequence-based protein language models for variant fitness prediction. Future investigations may refine these predictions through alternative scoring methods or supervised fine-tuning of experimental data. Ultimately, integrating this computational framework with orthogonal experimental approaches may enhance our understanding of germline variant interactions and their role in cancer susceptibility and progression.

## Data Availability

The molecular dynamics simulation data, including the equilibrated trajectories without water molecules, is accessible on Zenodo at DOI: 10.5281/zenodo.14008334.

## Supporting Information Available

Full sequence of wild type EXO5, and its secondary structure; RMSD plots; secondary structure of the EXO5 proteins over the MD simulation; change in the PyDSSP-assigned secondary structure of the α4 region; clustering results of the EXO5 backbone dihedrals; contact maps with the occurrence of interactions between residues of the NLS and the rest of EXO5; RMSD of the NLS (residues 197–215) across all replicas; and analysis of the protein–ssDNA interface (PDF)
PLM score analysis details (XLSX)
