# Supplementary material for: Integrative Computational Analysis of Common EXO5 Haplotypes: Impact on Protein Dynamics, Genome Stability, and Cancer Progression
Source: J Chem Inf Model. 2025 Mar 21;65(7):3640–54. doi: 10.1021/acs.jcim.5c00067 (PMC12004521; doi:10.1021/acs.jcim.5c00067)
Supplement: Supplementary file 1 — ci5c00067_si_001.pdf [file ci5c00067_si_001.pdf]

Supporting Information
for

An Integrative Computational Analysis of Common EXO5 Haplotypes: Impact on Protein Dynamics, Genome Stability, and Cancer Progression

Fabio Mazza[†], Davide Dalfovo[†], Alessio Bartocci[‡¶], Gianluca Lattanzi[‡¶§] and Alessandro Romanel[*†§]

[†]Department of Cellular, Computational and Integrative Biology (CIBIO), University of Trento, Via Sommarive 9, 38123,Trento, Italy
[‡]Department of Physics, University of Trento, Via Sommarive 9, 38123,Trento, Italy
INFN-TIFPA, Trento Institute for Fundamental Physics and Applications, Via Sommarive 14, 38123, Trento, Italy
[§]Co-senior authorship.
E-mail: alessandro.romanel@unitn.it

```
                  10        20        30        40        50        60        70        80
          ---------+---------+---------+---------+---------+---------+---------+---------+
 UniProt: MAETREEETVSAEASGFSDLSDSEFLEFLDLEDAQESKALVNMPGPSSESLGKDDKPISLQNWKRGLDILSPMERFHLKY
          ................................................................................
PDB 7LW9: --------------------------------------------------------------------ILSPMERFHLKY
```

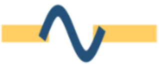

```
        90       100       110       120       130       140       150       160       170
---------+---------+---------+---------+---------+---------+---------+---------+---------+
LYVTDLATQNWCELQTAYGKELPGFLAPEKAAVLDTGASIHLARELELHDLVTVPVTTKEDAWAIKFLNILLLIPTLQSEGHIREFPVFG
..........................XXXXXXXXXXXXXXXXXXXXXXXXXX......................................
LYVTDLATQNWCELQTAYGKELPGFL--------------------------TVPVTTKEDAWAIKFLNILLLIPTLQSEGHIREFPVFG
```

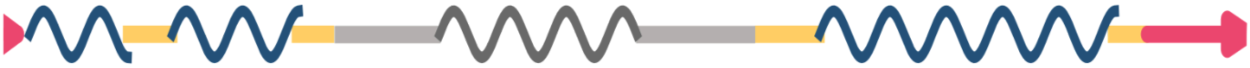

```
       180       190       200       210       220       230       240       250       260
---------+---------+---------+---------+---------+---------+---------+---------+---------+
EGEGVLLVGVIDELHYTAKGELELAELKTRRRPMLPLEAQKKKDCFQVSLYKYIFDAMVQGKVTPASLIHHTKLCLEKPLGPSVLRHAQQ
.X........................................................................................
EVEGVLLVGVIDELHYTAKGELELAELKTRRRPMLPLEAQKKKDCFQVSLYKYIFDAMVQGKVTPASLIHHTKLCLEKPLGPSVLRHAQQ
```

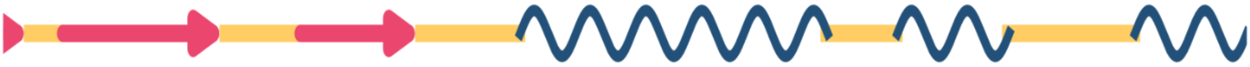

```
       270       280       290       300       310       320       330       340       350
---------+---------+---------+---------+---------+---------+---------+---------+---------+
GGFSVKSLGDLMELVFLSLTLSDLPVIDILKIEYIHQETATVLGTEIVAFKEKEVRAKVQHYMAYWMGHREPQGVDVEEAWKCRTCTYAD
..........................................................................................
GGFSVKSLGDLMELVFLSLTLSDLPVIDILKIEYIHQETATVLGTEIVAFKEKEVRAKVQHYMAYWMGHREPQGVDVEEAWKCRTCTYAD
```

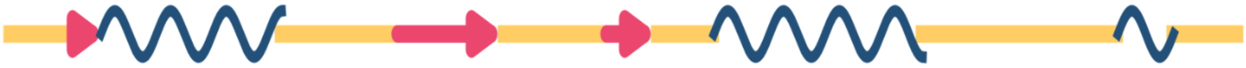

```
       360       370
---------+---------+---
ICEWRKGSGVLSSTLAPQVKKAK
.......................
ICEWRKG----------------
```


***Figure S1**: Full sequence of the WT EXO5 protein aligned to the sequence of the crystallographic structure found in PDB 7LW9. The secondary structure of the experimental structure is shown in color, while the structure predicted by AlphaFold2 is in grey.*

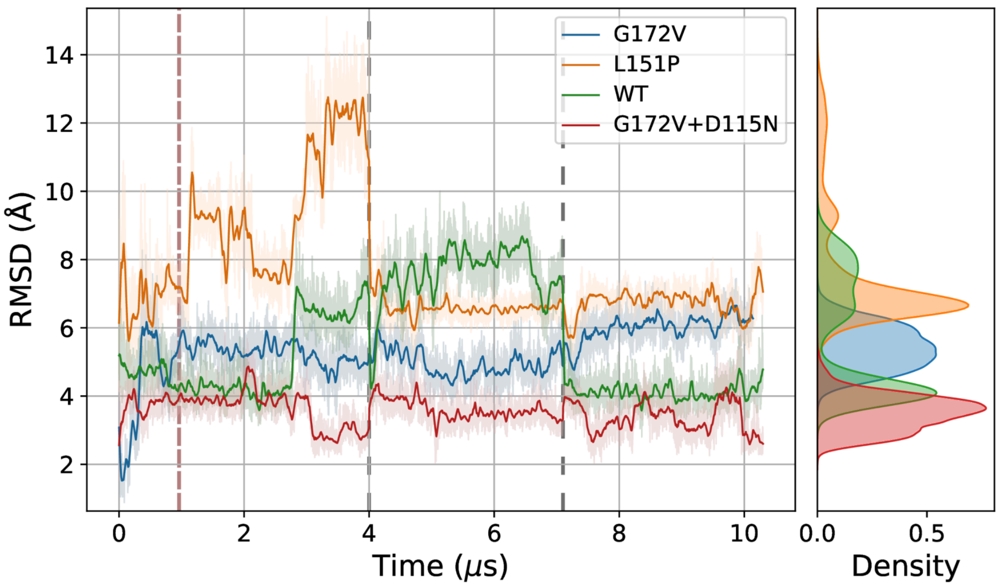

Figure S2: *RMSD of residues 106-138 along the MD simulations. Frames aligned to the whole initial EXO5 structure. Lighter shades are the raw data, calculated every 0.5 ns, while the darker lines are a moving average with a 40 ns uniform window. The dashed red line indicates the equilibration cutoff, while the dashed gray lines separate different replicas.*

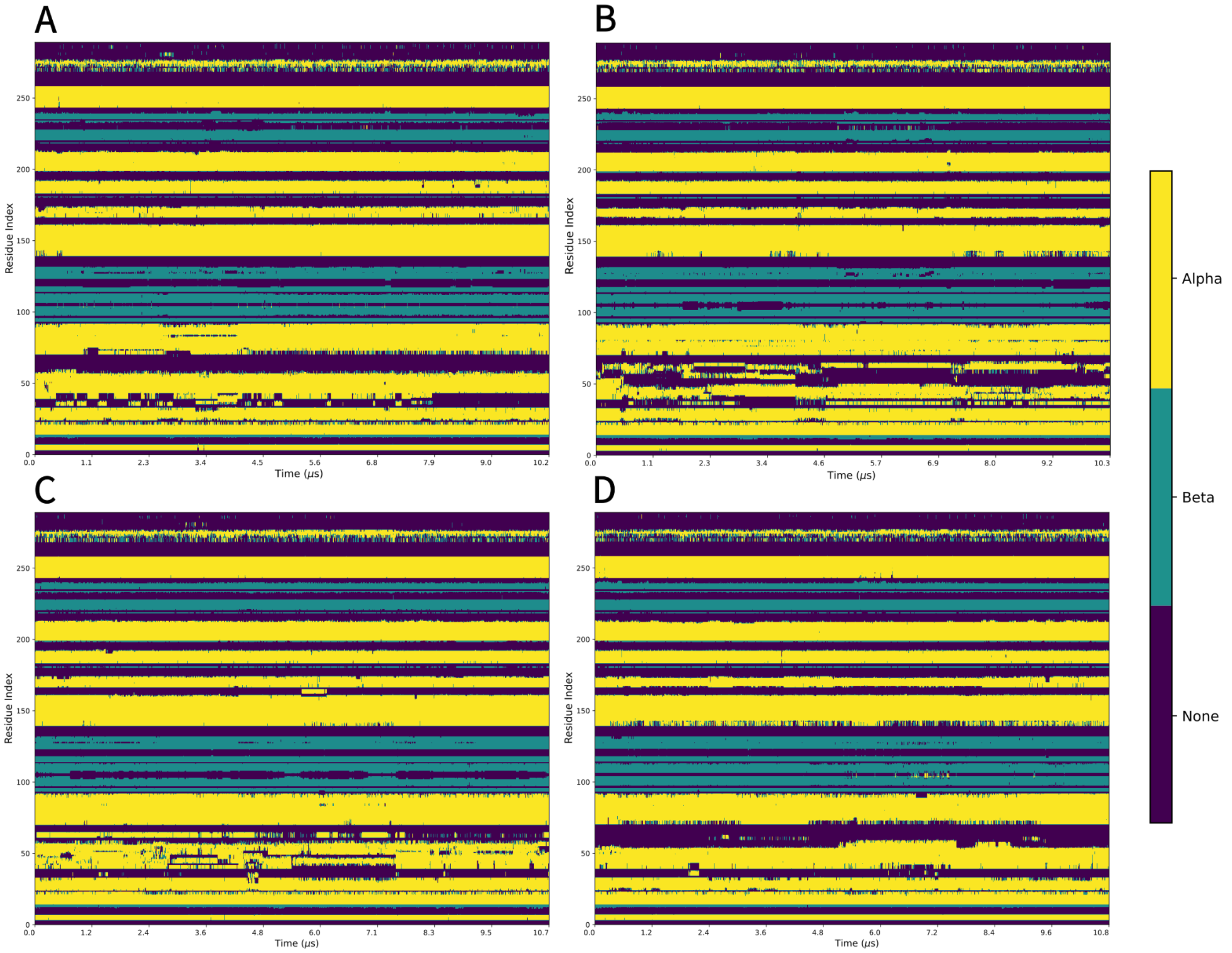

Figure S3*: Secondary structure of the EXO5 proteins over the MD simulation replicas, as determined by MDAnalysis using PyDSSP: G172V EXO5 (A), L151P EXO5 (B), WT EXO5 (C), G172V+D115N EXO5 (D). Note that residue indices are shifted by -68 compared to the reference sequence used in the main text.*

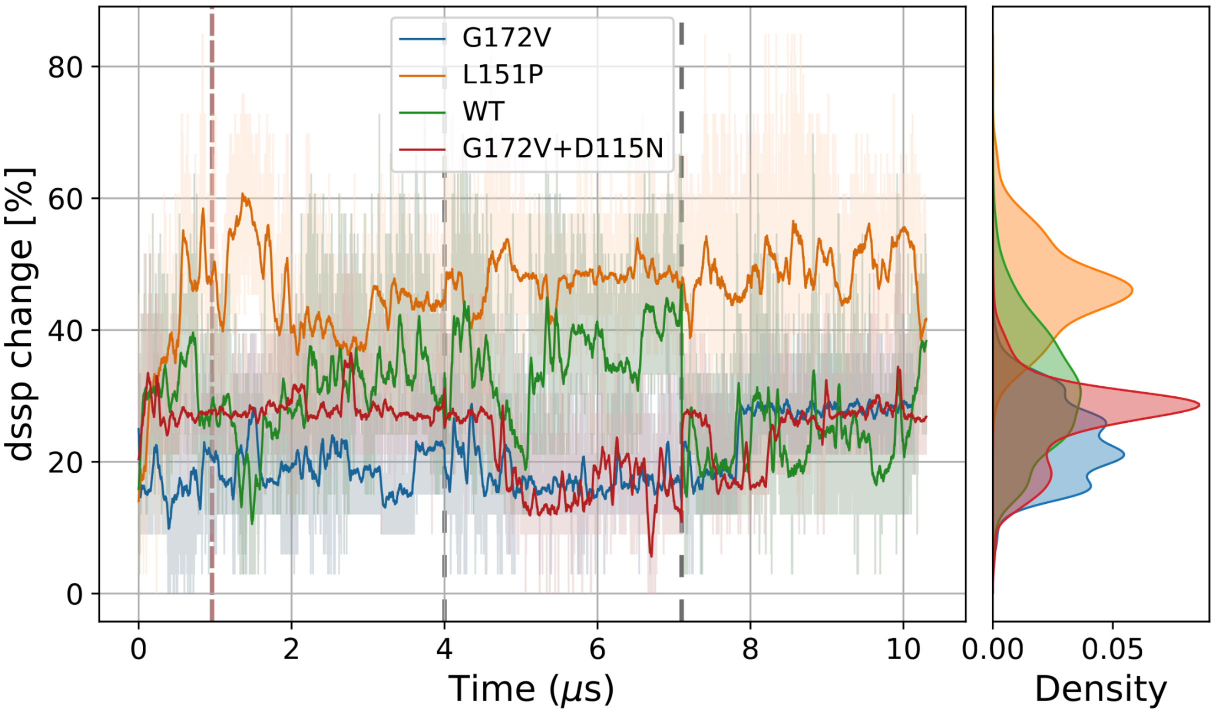

Figure S4*: Change in the PyDSSP-assigned secondary structure of the α4 region. Lighter shades are the raw data, calculated every 0.5 ns, while the darker lines are a moving average with a 40 ns uniform window. The dashed red line indicates the chosen equilibration cutoff, while the dashed grey lines separate different replicas.*

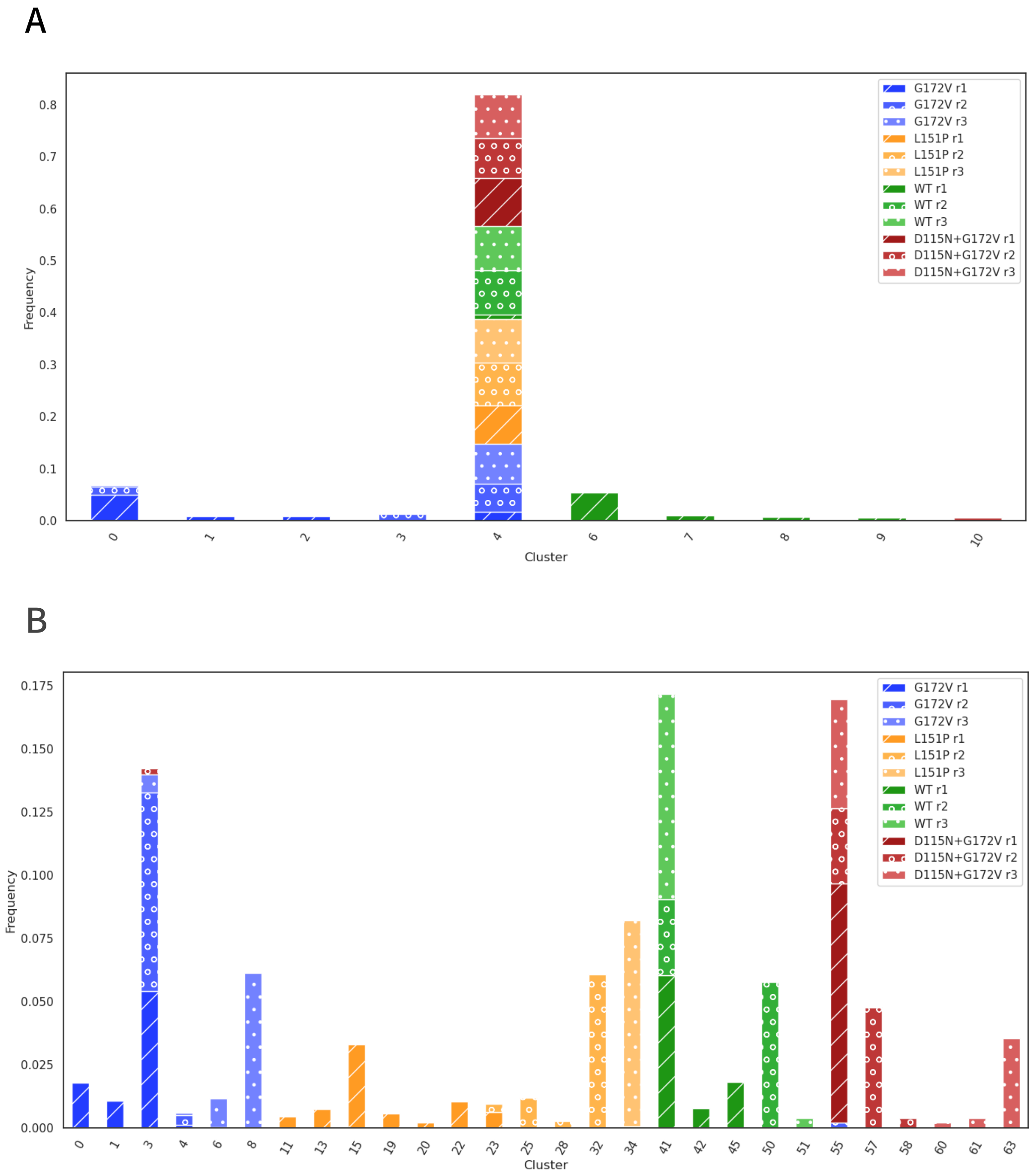

Figure S5: *Backbone dihedral clustering results of the EXO5 structures excluding the alpha4 region (A) and in the alpha4 region (B). Obtained using Advanced Density Peaks from the DADApy package, with Z=3, halo=False (A) and Z=5 (stricter condition to classify two clusters as separate), halo=False (B). Clusters containing less than 0.5% of frames are not shown.*

Clusters 3, 8, 15, 32, 34, 41, 50, 55, 57 and 63 are the ones shown in Figure 4B of the main text.

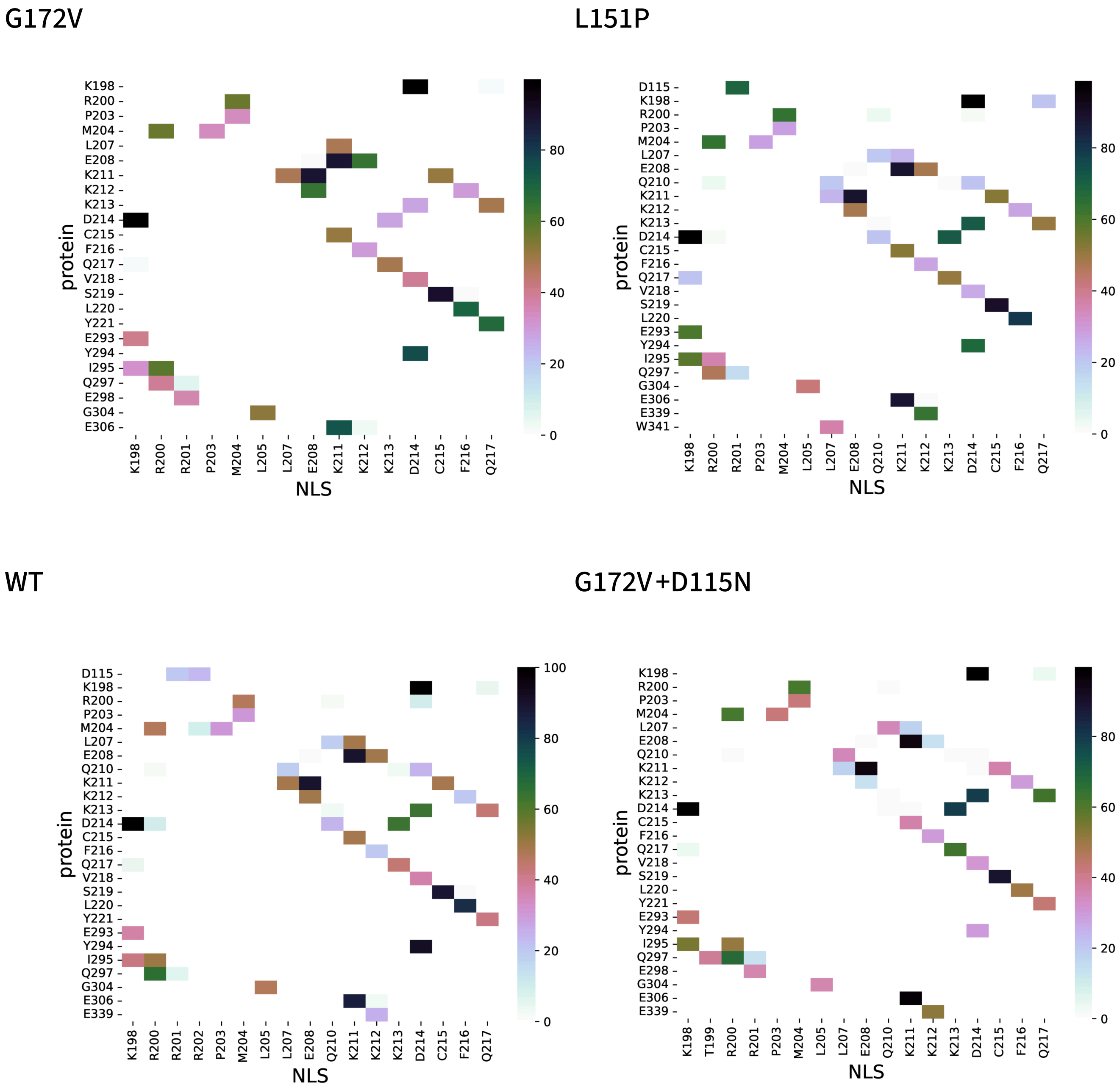

Figure S6: *Contact maps with the occurrence of interactions between residues of the Nuclear Localization signal and the whole EXO5 protein, averaged across all replicas. Contacts with less then 20% of occurrence are not shown.*

A

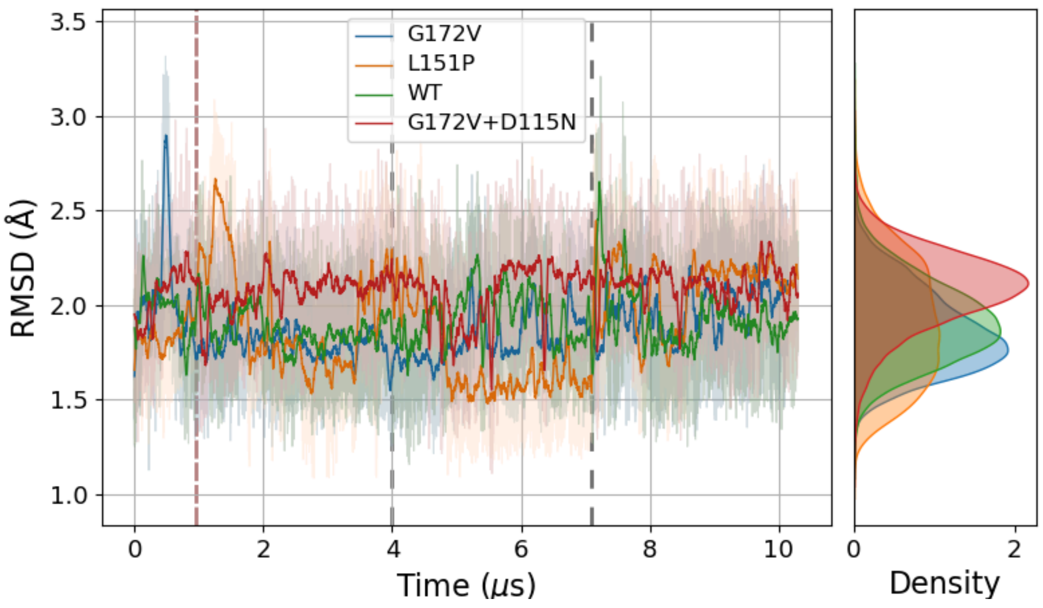

B

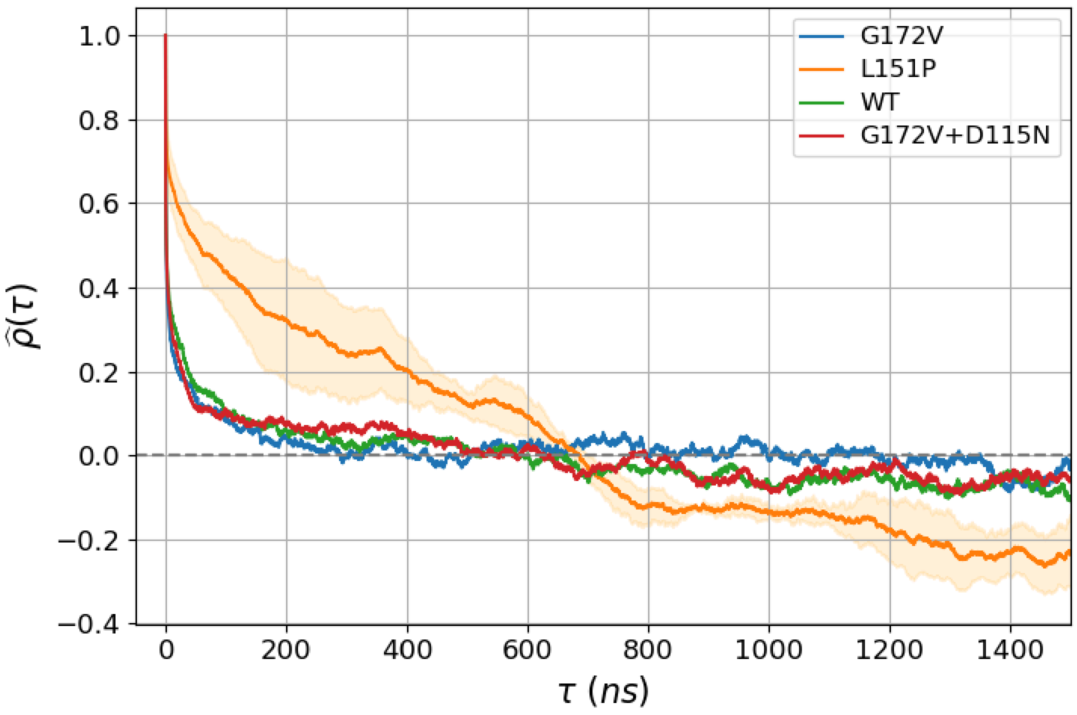

Figure S7: *RMSD of the Nuclear Localization Signal (residues 197-215) across all replicas (A). Autocorrelation function of the RMSD of the NLS, obtained by averaging the autocorrelation functions computed separately for each replica (B). For L151P EXO5, the standard error of the mean is shown as the light orange shade. Errors for the other structures are not shown for clarity.*

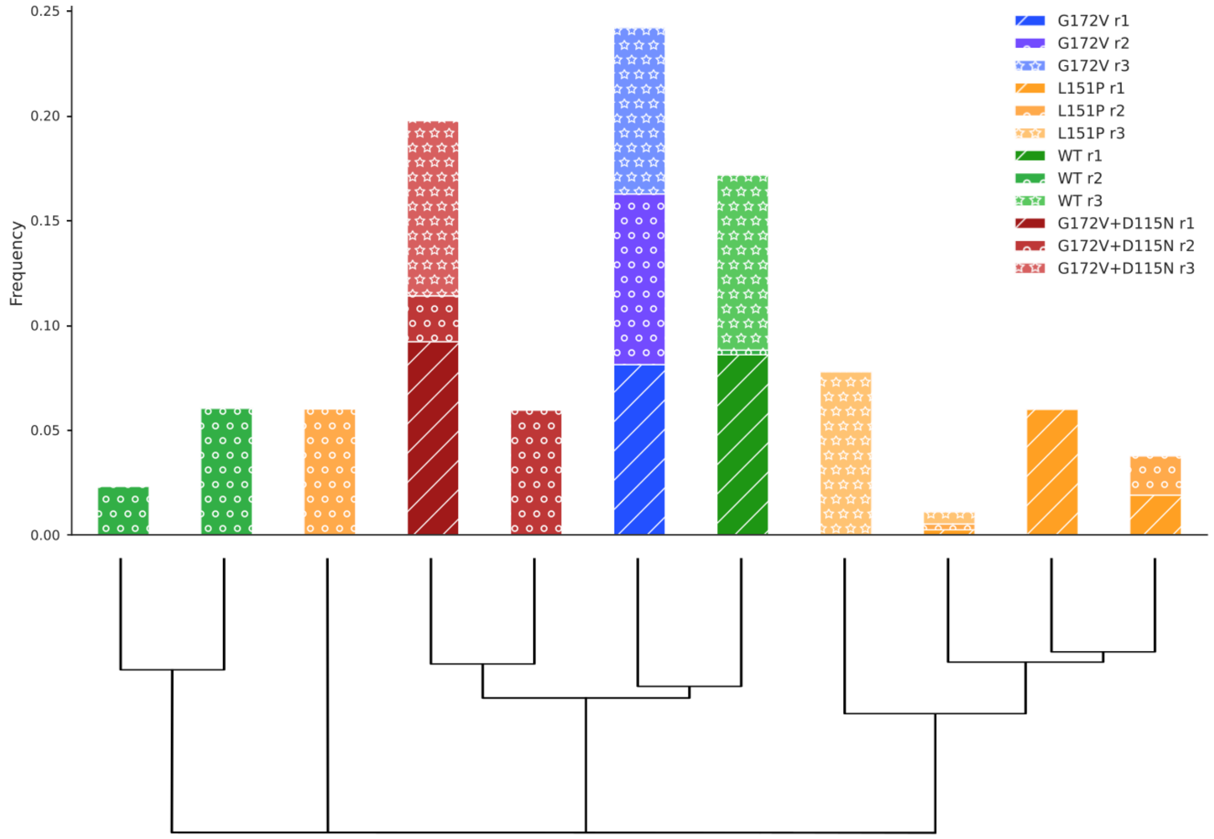

Figure S8: *Clustering results for the contact map of the protein-DNA interface. On top the histogram with the cluster distribution, on the bottom the dendrogram showing the hierarchy of the clusters. Performed with the Advanced Density Peaks algorithm with the cluster separation parameter Z=5.*
